# Identification of differentially expressed mRNA/lncRNA modules in acutely regorafenib-treated sorafenib-resistant Huh7 hepatocellular carcinoma cells

**DOI:** 10.1371/journal.pone.0301663

**Published:** 2024-04-11

**Authors:** Mina Baek, Minjae Kim, Hae In Choi, Bert Binas, Junho Cha, Kyoung Hwa Jung, Sungkyoung Choi, Young Gyu Chai

**Affiliations:** 1 Department of Molecular and Life Science, Hanyang University, Ansan, Republic of Korea; 2 Department of Applied Artificial Intelligence, Hanyang University, Ansan, Republic of Korea; 3 Department of Biopharmaceutical System, Gwangmyeong Convergence Technology Campus of Korea Polytechnic II, Incheon, Republic of Korea; 4 Department of Mathematical Data Science, Hanyang University, Ansan, Republic of Korea; Hawler Medical University, IRAQ

## Abstract

The multikinase inhibitor sorafenib is the standard first-line treatment for advanced hepatocellular carcinoma (HCC), but many patients become sorafenib-resistant (SR). This study investigated the efficacy of another kinase inhibitor, regorafenib (Rego), as a second-line treatment. We produced SR HCC cells, wherein the PI3K-Akt, TNF, cAMP, and TGF-beta signaling pathways were affected. Acute Rego treatment of these cells reversed the expression of genes involved in TGF-beta signaling but further increased the expression of genes involved in PI3K-Akt signaling. Additionally, Rego reversed the expression of genes involved in nucleosome assembly and epigenetic gene expression. Weighted gene co-expression network analysis (WGCNA) revealed four differentially expressed long non-coding RNA (DElncRNA) modules that were associated with the effectiveness of Rego on SR cells. Eleven putative DElncRNAs with distinct expression patterns were identified. We associated each module with DEmRNAs of the same pattern, thus obtaining DElncRNA/DEmRNA co-expression modules. We discuss the potential significance of each module. These findings provide insights and resources for further investigation into the potential mechanisms underlying the response of SR HCC cells to Rego.

## Introduction

Hepatocellular carcinoma (HCC) is the most common primary liver cancer and closely associated with chronic liver disease and cirrhosis [1]. An important first-line treatment for HCC is sorafenib [2], a multi-targeted tyrosine kinase inhibitor that suppresses cell proliferation and angiogenesis in advanced HCC. However, most patients become sorafenib-resistant (SR) within 6 months of treatment [3], leading to the need for second-line treatment options such as regorafenib (Rego) [4, 5]. However, the molecular mechanisms of SR and Rego remain poorly understood, hindering the further improvement of treatment options.

Long non-coding RNAs (lncRNAs) are a class of RNA molecules that are over 200 nucleotides in length and do not encode proteins [6, 7]. They play critical roles in the development and progression of various cancer types by regulating gene expression, signaling pathways, and epigenetic modifications [8]. LncRNAs have also been identified as potential targets to overcome drug resistance in cancer cells [9], and recent research demonstrated that targeting specific lncRNAs can enhance the efficacy of cancer treatment by sensitizing drug-resistant cancer cells [10]. The role of lncRNAs has also been explored specifically in SR HCC cells. For instance, MALAT1 [11] and NEAT1 [12] were found to be upregulated in HCC cells with acquired SR, and their knockdown resulted in re-sensitization to sorafenib. Similarly, SNHG1 was upregulated in SR HCC cells, and its knockdown improved sorafenib sensitivity by promoting apoptosis and suppressing cell migration [13]. These studies demonstrate the involvement of lncRNAs in the development of drug resistance in cancer cells, including SR HCC cells, and suggest that targeting specific lncRNAs may be a promising strategy to overcome SR.

The goal of the present study was to perform a comparative transcriptome profiling of the SR and Rego-treated SR (SR+Rego) HCCs, with the hope of identifying potential molecular targets to overcome SR. We first identified differentially expressed mRNAs (DEmRNAs) and lncRNAs (DElncRNAs). Using bioinformatics tools, we then identified pathways and functional categories that are shared by both sample types and that are enhanced or diminished in the (SR+Rego) samples.

## Materials and methods

### Cell culture and treatments

The HCC cell line Huh7 was purchased from the Korean Cell Line Bank (Seoul, Korea) and cultured in MEM supplemented with 10% heat-inactivated FBS, penicillin, and streptomycin (Thermo Fisher Scientific, Waltham, MA, USA) at 37°C in a humidified atmosphere with 95% air and 5% CO2. (Thermo Scientific, HERAcell 240, MA, USA) To establish SR, the cells were cultured for ~6 months in the presence of sorafenib, starting at 1 μM and with increments of 0.25 μM per passage until 5 μM. In order to assess acute drug effects on regular Huh7 cells (Con) or SR Huh7 cells, the cells were freshly seeded into the respective conditions as described in the subsections further below.

### Analyses of apoptosis and necrosis

Apoptosis and necrosis were determined using Annexin V FITC (BD Biosciences, Heidelberg, Germany) and propidium iodide (PI) (Sigma Aldrich, St. Louis, USA) labeling and flow cytometry. Briefly, Huh7 cells (Con) or SR Huh7 were seeded at a density of 5 x 103 cells per well in 6-well plates. After 24 hours, sorafenib was added at 5 μM, and the cells were incubated for an additional 48 hours. The cells were then harvested (5 x 105 cells) and washed twice (180 g, 10 minutes, and 4°C) with PBS. Each cell pellet was re-suspended in 100 μl of binding buffer (1×) and 5 μl of Annexin V FITC were added. After an incubation time of 10 minutes at room temperature, an additional 400 μl of binding buffer were added for a final volume of 500 μl. Cells were stained with PI (0.6 μg/ml) immediately before measurement. For compensation, unstained Huh7 cells were prepared and set at zero. Then, single-stained Huh7 cells (stained with either Annexin V FITC or PI) were analyzed to adjust the location of double-labeled cells. Unstained and single-stained controls were included in each experiment. Flow cytometry analyses were performed using FACSCalibur (Becton and Dickinson, Heidelberg, Germany) and data thus obtained were analysed with CellQuest software (Becton and Dickinson, Heidelberg, Germany).

### Cell viability assay

Cell viability was determined by using a PreMix water-soluble tetrazolium-1 (WST-1) cell proliferation assay kit (Takara, Shiga, Japan). Huh7 cells were seeded in 96-well plates at a density of 5 x 103 cells per well and treated with sorafenib for 0 to 24 hours. After treatment, PreMix WST-1 was added to each well, and the plates were incubated for 4 hours at 37°C. Absorbance was measured at 450 nm using a microplate reader, and the data represent three independent experiments (n = 3).

### Colony-formation assay

For colony formation assays, Huh7 cells (Con) or SR cells were washed and seeded at a density of 5 x 103 cells per well in 96-well plates. After 24 hours of incubation, DMSO or sorafenib (1, 5, or 10 μM) were added, and the cells were incubated for 7–10 days, with media changed every three days. When colonies had grown to a sufficient size, the cells were fixed with 4% paraformaldehyde (in PBS) and stained with a 0.05% crystal violet solution before being imaged. The colony formation assay was performed in biological duplicates for each cell type and drug combination.

### Cell cycle analysis

Cells were seeded at a density of 5 x 103 cells per well in 6-well plates. After 24 hours of incubation, sorafenib was added at a concentration of 5 μM, and the cells were incubated for 48 hours. Cells were collected twice in PBS and fixed in 75% ethanol, followed by incubation with 1 mg/ml Ribonuclease A (Sigma-Aldrich, St. Louis, USA) for 30 minutes at 37°C. Subsequently, cells were washed twice in PBS and stained with PI. Prior to flow cytometry, cells were filtered through a 70 μM cell strainer. Flow cytometry analysis was performed on a Becton Dickinson FACSCalibur device (BD Biosciences, San Jose, CA, USA).

### RNA library preparation and RNA-seq

Total RNA was extracted from 12 independent samples of Huh7 cells divided into 4 groups: DMSO-treated Huh7 cells (Con, n = 3), regorafenib-treated Huh7 cells (Rego, n = 3), sorafenib-resistant Huh7 cells (SR, n = 3), and regorafenib (5 μM)-treated sorafenib-resistant Huh7 cells (SR+Rego, n = 3). Where added, DMSO or regorafenib were present for the last 24 hours preceding the RNA extraction. The RNA library was created as previously described [14]. Briefly, RNA was extracted using RNAiso Plus (Takara, Shiga, Japan) and the RNeasy Mini Kit (QIAGEN Inc., Hilden, Germany), and rRNA was depleted using the RiboMinus Eukaryote kit (Invitrogen, Carlsbad, CA, USA). RNA libraries were created using the NEBNext Ultra Directional RNA Library Preparation Kit for Illumina (New England BioLabs, Ipswich, MA, USA), and transcriptome sequencing was performed using the Illumina HiSeq2500 platform (Macrogen, Seoul, Korea).

### Identification of differentially expressed lncRNAs and mRNAs

To identify differentially expressed mRNAs (DEmRNAs) and long non-coding RNAs (DElncRNAs), a previously reported pipeline [15] was followed, which included a comprehensive reference list of known mRNAs and lncRNAs. Briefly, after quality control and trimming of the FASTQ files with Trimmomatic (version 0.36) [16], the resulting high-quality reads were aligned to the GENCODE Homo sapiens reference sequence GRCh38 (Release 27) using the STAR (version 2.7.8) alignment software [17]. The sequencing depth and RNA composition were adjusted to normalize DElncRNAs and DEmRNAs using the median method with default parameters of DESeq2 (version 2.15) [18]. The DESeq2 R package was used to conduct differential expression analysis of lncRNAs and mRNAs. The DElncRNAs and DEmRNAs were selected with a cutoff of log2 fold change (log2 FC) ≥ |±2.0|, and false discovery rate (FDR) ≤ 0.05. The RNA-seq data were deposited in the Gene Expression Omnibus database under dataset accession number GSE242333.

### Functional annotation and canonical pathway analysis

Database for Annotation, Visualization, and Integrated Discovery (DAVID, version 6.8) software (http://david.abcc.ncifcrf.gov/home.jsp) was used to analyze the biological functions in the datasets [19]. The gene ontology (GO) enrichment was examined using a modified Fisher’s exact *p*-value, and a FDR < 0.05 was set as the threshold. We also used the KEGG Orthology Based Annotation System (KOBAS, version 3.0) software (http://kobas.cbi.pku.edu.cn/) [20] to identify enriched KEGG pathways in the datasets, with an FDR < 0.05 used as the cutoff for KEGG pathway enrichment analysis.

### Weighted gene co-expression network analysis (WGCNA)

First, the Pearson correlation coefficient (*r*) values were calculated to assess the similarity of the expression patterns of transcripts. Then, a scale-free network was obtained by weighting the correlation coefficient between transcripts with soft-thresholding power. A module is defined as a cluster of densely interconnected transcripts in terms of co-expression. We considered an |*r|*≥ 0.75 as a meaningful value. The Cytoscape MCODE plug-in (Version 3.4.0, available online: http://www.cytoscape.org/) [21] was applied for visualization of the co-expression networks.

Gene co-expression networks were constructed using the WGCNA R package. First, a soft-thresholding power value was determined in order to achieve a scale-free topology for the co-expression network. Then, a weighted adjacency matrix was constructed using a power function, and genes were hierarchically clustered based on their topological overlap measure (TOM). Modules of co-expressed genes were identified using the dynamic tree-cutting algorithm. Module eigengenes, which are the first principal component of each module, were calculated to represent the overall gene expression level of each module. The correlation between module eigengenes and phenotypic traits was calculated using the ‘cor’ function in R. Pearson’s correlation coefficient was used to measure the strength and direction of the association between each module eigengene and each trait. The significance of the correlations was assessed using a permutation test, and a Bonferroni correction was applied to adjust for multiple testing.

### Quantitative reverse transcription PCR (qRT-PCR)

The total RNA was extracted and cDNA was prepared following the manufacturer’s instructions (Takara, Shiga, Japan). Quantitative Reverse Transcription PCR (qRT-PCR) was carried out using an ABI 7500 real-time PCR system (Applied Biosystems Inc., Foster City, CA, USA). The expression levels were normalized using the internal control, glyceraldehyde-3-phosphate dehydrogenase (GAPDH), and analyzed using the comparative critical threshold (ΔΔCT) method. Specific primers were designed using Primer Bank (http://pga.mgh.harvard.edu/primerbank/index.html), and the primer sequences used for qRT-PCR are listed in S1 Table.

### Biofunctional analysis

To investigate the genetic networks and regulatory effects of regorafenib, the analysis focused on genes from RNA-seq datasets that met two criteria: log2 FC ≥ |±2.0|, and FDR ≤ 0.05 in both regorafenib-treated Con and SR cells. Subsequently, each of these selected genes was subjected to mapping through Ingenuity Pathway Analysis (IPA; QIAGEN Inc., Hilden, Germany, https://www.qiagenbioinformatics.com/products/ingenuitypathway-analysis). The IPA software performs functional analysis, revealing the involvement of genes in various biological functions and diseases.

### Statistical analyses

The statistical analysis of the qRT-PCR data was carried out using IBM SPSS Statistics ver. 26.0 (IBM Corporation, Armonk, NY, USA). A one-way ANOVA was performed, followed by Tukey’s honestly significant difference (HSD) post hoc test. Differences with a *p*-value of ≤ 0.05 were considered statistically significant.

## Results

### Establishment and verification of SR HCC cells

**Fig 1:**
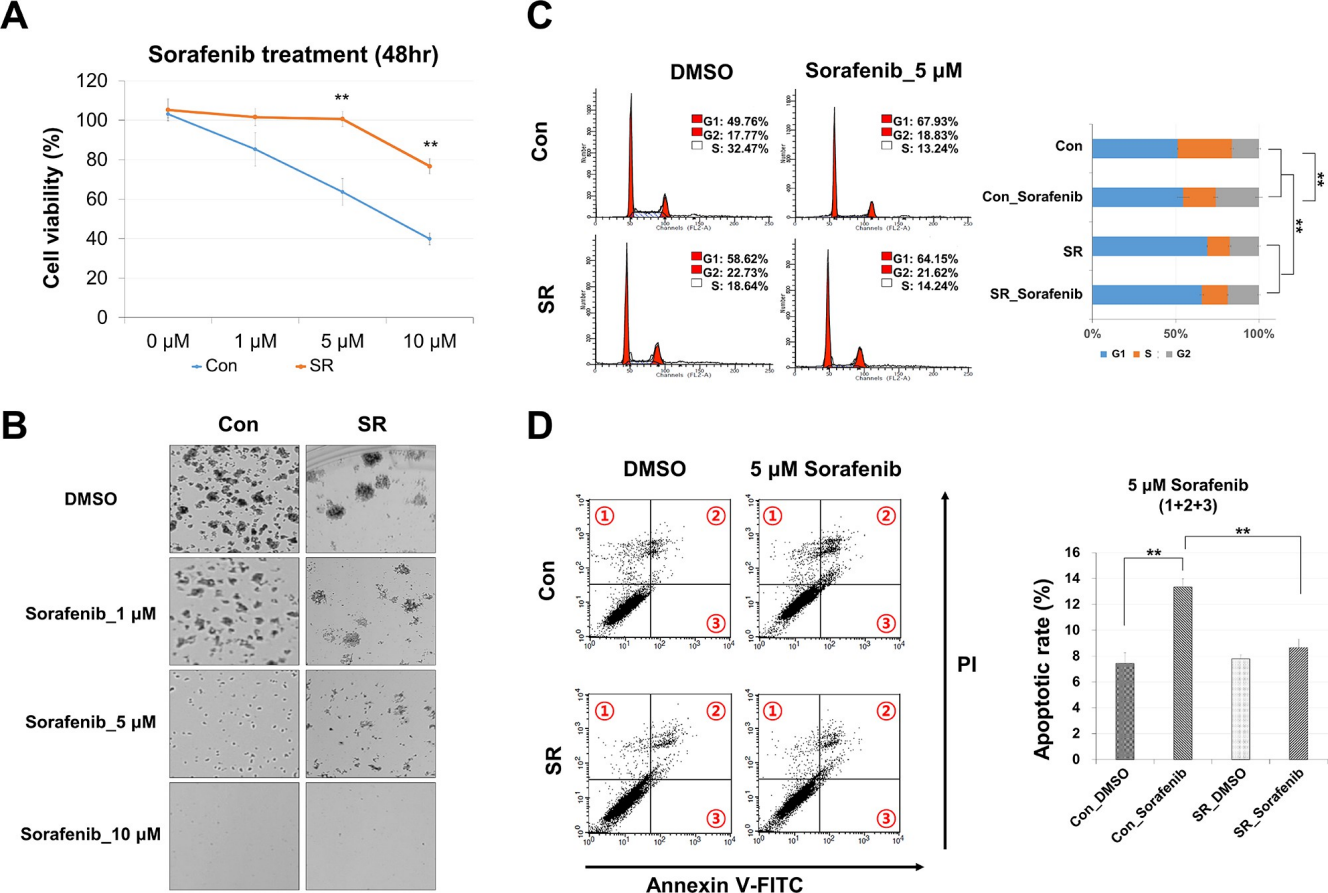
Establishment and confirmation of SR HCC cells. (A) Cell viability analysis of regular Huh7 Con cells and Huh7 SR cells, both treated for 48 hours with either vehicle (DMSO) or the indicated concentrations of sorafenib (0–10 μM). (B) Colony formation assays of Con and SR cells performed in the presence of vehicle or sorafenib. Representative images of cell colonies are shown. (C) Cell-cycle distribution and (D) apoptosis analyses of Con and SR cells treated with vehicle or 5 μM sorafenib for 48 hours. Flow cytometry was used to analyze cell-cycle distribution and apoptosis. Data are presented as mean ± standard deviation (SD) (*p ≤ 0.05 and **p ≤ 0.01).

The study was initiated by the establishment of a sorafenib-resistant (SR) HCC line. In this regard, Huh7 cells were chronically exposed to increasing concentrations of sorafenib, reaching a plateau at 5 μM (see Materials and Methods). Subsequently, drug resistance was quantified through viability and plating assays over 48 hours and ≥ 1 week, respectively. As expected, viability of the SR cells was compromised only when the sorafenib concentration exceeded 5 μM, while the original Huh7 cells showed a significant death rate already at 1 μM (Fig 1A). Likewise, colony formation by the SR cells was not compromised at up to 5 μM, but was largely lost at that concentration in the control cells (Fig 1B). On the other hand, compared to the untreated (DMSO) control cells, the SR cells exhibited a reduced plating efficiency (Fig 1B) and a smaller S-phase/larger G1-phase fraction regardless of the acute absence or presence of (up to 5 μM) sorafenib (Fig 1C). A similar S-phase reduction was caused by the acute treatment of the control cells, while no further reduction was seen in the SR cells (Fig 1C). Reduced cell viability and reduced colony formation may involve apoptosis. Indeed, acute sorafenib treatment almost doubled the apoptotic rate in the control cells (Fig 1D. In contrast, the apoptotic rate of the SR cells, which was the same as in the untreated (DMSO) control cells, was not significantly affected by the acute presence or absence of sorafenib (Fig 1D). The SR cells were protected from the apoptosis-inducing effects of sorafenib. It is less clear whether the same applies to the anti-proliferative effect, given that the acute and chronic exposures altered the cell cycle in the same way. Overall, chronic exposure to sorafenib established a resistant cell line (SR) with significantly reduced sensitivity to sorafenib.

### Identification of DEmRNAs and DElncRNAs

**Fig 2:**
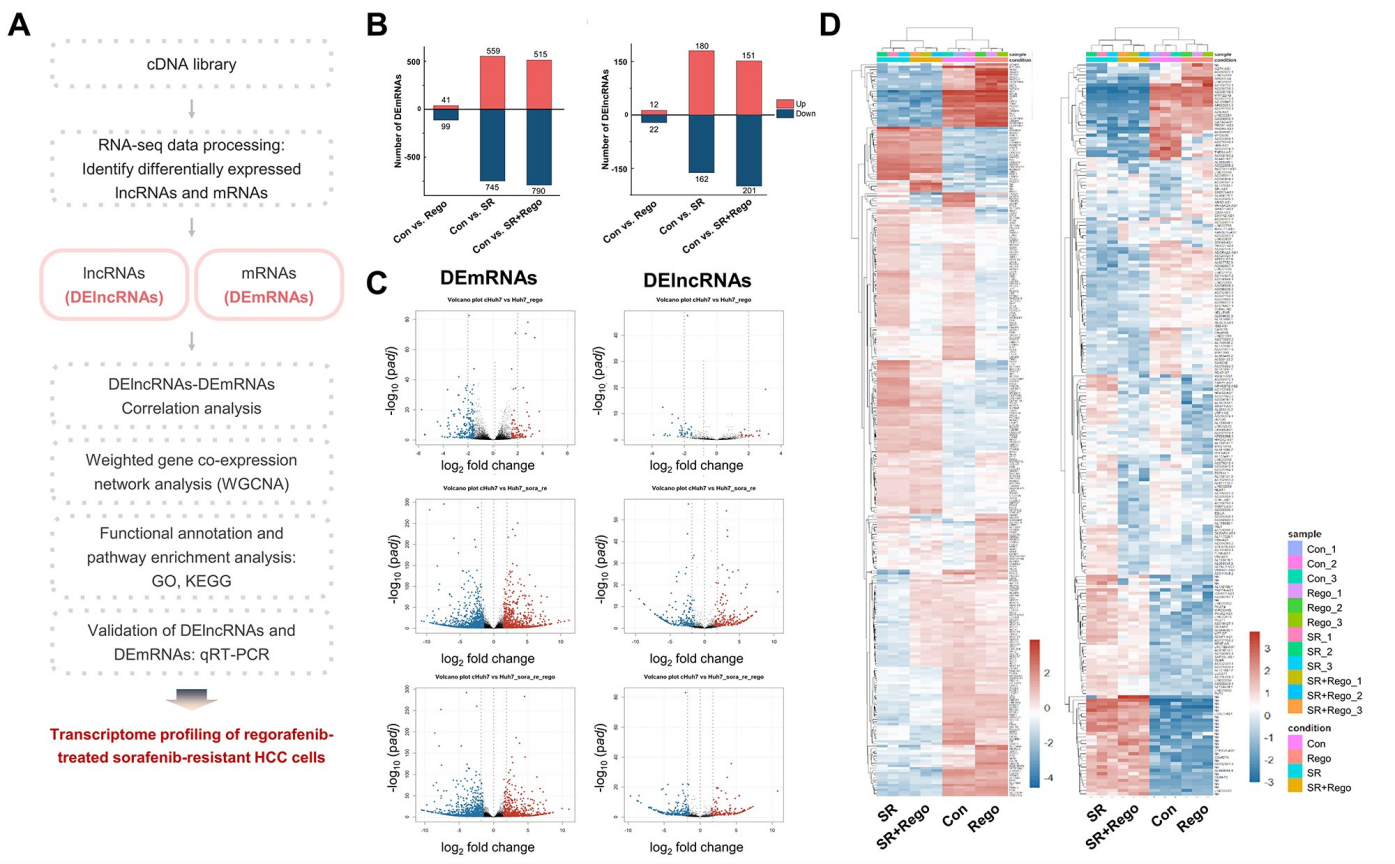
Identification of DEmRNAs and DElncRNAs in SR HCC. (A) Schematic diagram summarizing the project workflow. (B) Bar chart showing the number of up- and downregulated DEmRNAs and DElncRNAs in Con cells (left column) and SR cells (middle and right columns) that were acutely treated with Rego (columns “Rego” and “SR+Rego”) or with vehicle (“SR”); all in comparisons with Con. (C) Volcano plots illustrating the DEmRNAs (left panel) and DElncRNAs (right panel) in Rego-treated Con cells (upper panel), SR cells (middle panel), and SR+Rego cells (bottom panel). The gray vertical dot lines indicate log_2_ fold change (log_2_ FC) of ≥ |±2.0|. The gray horizontal dot lines indicate -log_10_ (FDR) ≤ 0.05. (D) Heat map displaying the DEmRNAs (left panel) and DElncRNAs (right panel) in Rego, SR, and SR+Rego cells identified by RNA-seq (log_2_ FC ≥ |±2.0|, and FDR ≤ 0.05). The color scale represents the log_2_ FC values. The data represent three independent experiments.

The study flow chart is depicted in Fig 2A. The following samples were gene expression-profiled by RNA-seq: Con cells, SR cells, Con cells acutely treated for 24 hours with 5 μM of Rego (Rego), and SR cells acutely treated for 24 hours with 5 μM of Rego (SR+Rego). The subsequent analysis revealed the differential expression of 1304 mRNAs and 342 lncRNAs in SR vs. Con cells with a cutoff log2 fold change (FC) of ≥|±2.0| and a false discovery rate (FDR) ≤0.05 (Fig 2B, column SR). Additionally, 1305 DEmRNAs and 352 DElncRNAs were identified in SR+Rego vs. Con cells (Fig 2B, column SR+Rego). The volcano plot (Fig 2C) and heat map (Fig 2D) demonstrate the expression patterns of the differentially up- and downregulated genes in SR and SR+Rego cells. The DEmRNAs and DElncRNAs of Rego, SR, and SR+Rego (each versus Con) are listed in S2–S7 Tables, respectively.

### Functional annotation and pathway enrichment analysis of DEmRNAs in SR and SR+Rego cells

Next, we conducted a comprehensive functional assessment utilizing Gene Ontology (GO) and Kyoto Encyclopedia of Genes and Genomes (KEGG) pathway analyses. The outcomes are presented in S1–S3 Figs, providing a detailed overview of the functional insights across all experimental groups. The GO analysis revealed biological process (BP) categories of commonly up- or down-regulated DEmRNAs in SR cells (SR vs. Con) and SR+Rego cells (SR+Rego vs. Con), presented in S4A and S4B Fig, while KEGG analysis is depicted in S4C and S4D Fig Distinct patterns of up- or down-regulated DEmRNAs emerged in SR cells (SR vs. Con) and SR+Rego cells (SR+Rego vs. Con), as shown in S5A and S5B Fig Corresponding KEGG analysis annotations for these comparisons are shown in S5C and S5D Fig. Additionally, functional annotation analysis of DElncRNAs was performed using GREAT, revealing their proximity to transcriptional start sites, which suggests potential regulatory roles for protein-coding genes (S6 and S7 Figs). Of significance, the DEmRNAs consistently upregulated in both the SR cells and SR+Rego cells exhibited significant enrichment in pathways encompassing cancer, PI3K-Akt signaling, focal adhesion, relaxin signaling, and TNF signaling pathways. Notably, we observed an increased activation of protein kinase B signaling (AKT) in response to regorafenib.

### Analysis of gene expression patterns

**Fig 3:**
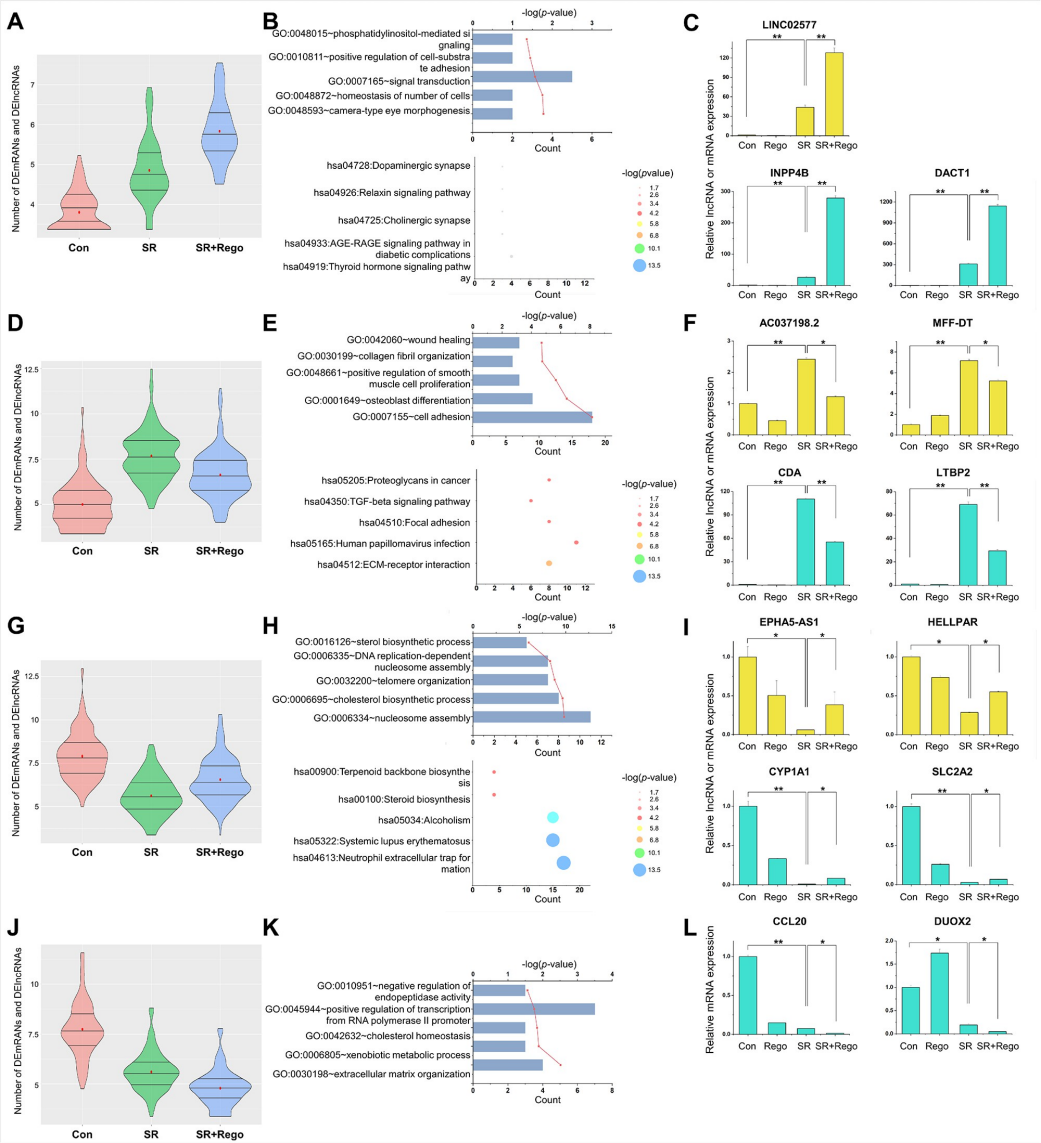
Gene expression pattern analysis of SR and SR+Rego cells. Violin plots showing differentially expressed (vs. Con) gene cohorts according to their type of change in SR vs. Con (left letter H or L) and SR+Rego vs. SR (right letter H or L). Specifically, (A) represents high-to-higher = HH; (D) represents high-to-lower = HL; (G) represents low-to-higher = LH, and (J) represents low-to-lower = LL. (B, E, H, K) GO BP terms (upper panels) and KEGG pathways (bottom panels) enriched in genes in each group. The columns indicate the count values of genes enriched in the GO term, and the red lines represent the -log_10_ (*p*-value) values. In the KEGG pathway enrichment analyses, the x-axis indicates the count value, and the size of the bubble represents the -log_10_ (*p*-value). (C, F, I, L) Differentially expressed mRNAs (turquoise) and lncRNAs (yellow) in each group were verified by qRT-PCR. The RNA levels were normalized to GAPDH and RNU6-1 (U6) transcript levels. The data represent three independent experiments, and the values are the mean ± SEM of triplicate experiments (*p ≤ 0.05 and **p ≤ 0.01).

Next, the patterns of differential gene expression were categorized with a two-letter code, where the first and second letter indicate the direction of the changed gene expression in SR vs. Con cells and in SR+Rego vs. SR cells, respectively. For example, HL would mean that the DEmRNAs and DElncRNAs of this category were expressed higher (H) in SR vs. Con but lower (L) in SR+Rego vs. SR. The DEmRNAs/DElncRNAs of each resulting group (HH, HL, LH, and LL) are listed in S8 Table. The DEmRNAs and DElncRNAs of the HH group (Fig 3A) were associated with GO terms such as cell homeostasis, signal transduction, and the KEGG pathway analysis showed enrichment of pathways related to thyroid hormone signaling and AGE-RAGE signaling in diabetic complications (Fig 3B and 3C). The GO terms of the HL group (Fig 3D) were mainly related to cell adhesion and osteoblast differentiation, and the KEGG pathway was enriched in pathways related to ECM-receptor interaction (Fig 3E). In the LH group (Fig 3G), the GO terms were mainly related to nucleosome assembly and cholesterol biosynthetic process, and the KEGG pathway was enriched in pathways related to neutrophil extracellular trap formation (Fig 3H). Finally, the GO terms of the LL group (Fig 3J) were mainly related to extracellular matrix organization and positive regulation of transcription from RNA polymerase II promoter, while the KEGG pathway did not show significant results (Fig 3K). Most of the identified DEmRNAs and DElncRNAs belonged to the HL and LH groups, meaning that their SR-related expression changes were reversed by regorafenib. The four groups that were identified from the RNA-seq data were validated by using qRT-PCR, which demonstrated a high level of agreement for both the DEmRNAs and DElncRNAs (Fig 3C,3F,3I and 3L.

### Identification of weighted gene co-expression network analysis (WGCNA) modules

**Fig 4:**
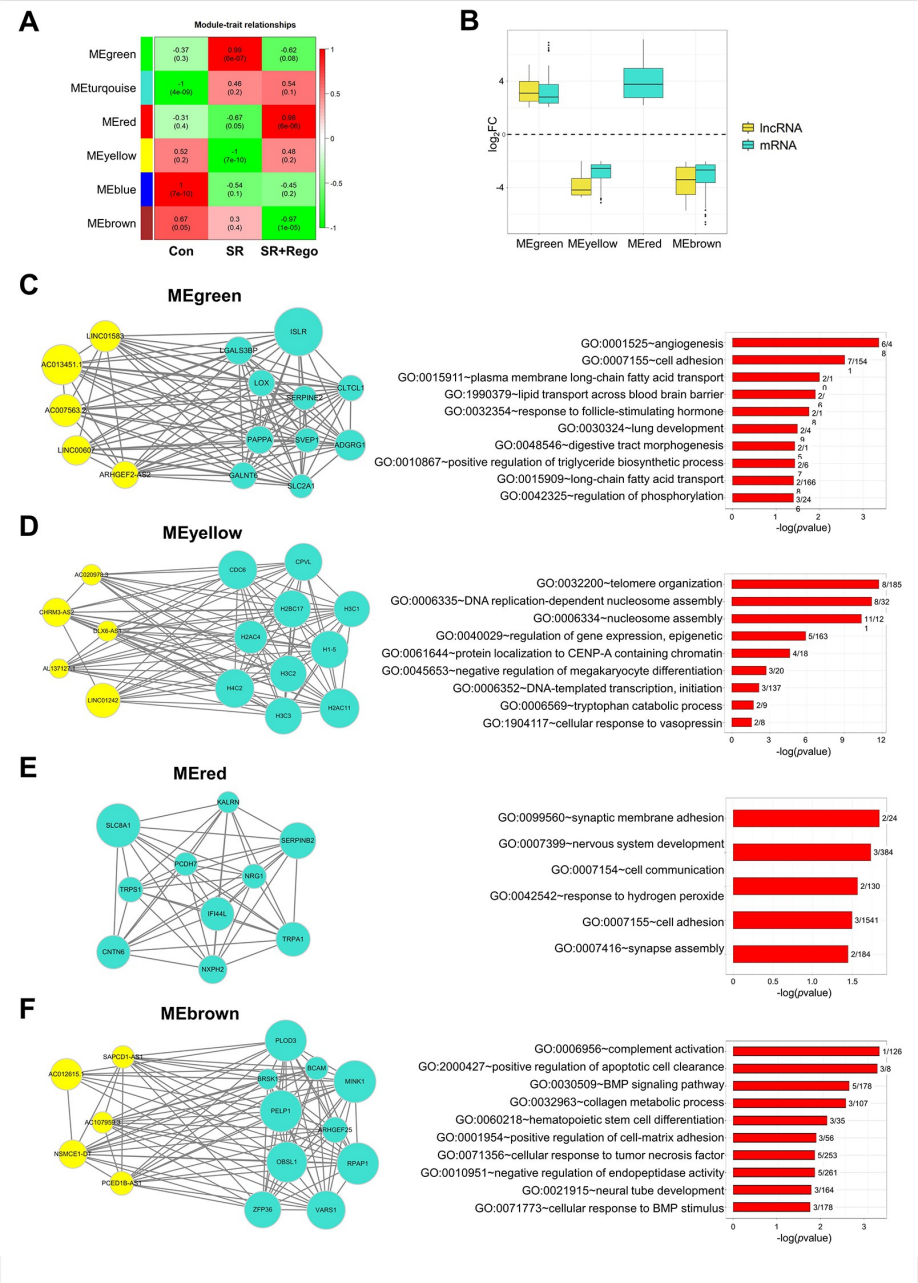
WGCNA analysis of DEmRNAs and DElncRNAs. Module-trait associations of DEmRNAs and DElncRNAs (A) were evaluated by correlations between control, SR, or SR+Rego traits. Pearson’s correlation coefficient (PCC) values and Student asymptotic *p*-values are displayed in the brackets. Box plots (B) show the expression fold changes of DEmRNAs and DElncRNAs in each module. (C-F) Co-expression networks and enriched GO BP terms are shown for selected DElncRNAs and their highly correlated DEmRNAs in four modules: MEgreen (C), MEyellow (D), MEred (E), and MEbrown (F). Yellow circles indicate DElncRNAs, and turquoise circles indicate DEmRNAs. The size of the circle represents the log_2_ fold change (log_2_ FC) value.

To delve deeper into the underlying mechanisms, WGCNA [22] was applied to all expressed lncRNAs and mRNAs. Module trait associations were obtained and then DEmRNAs and DElncRNAs previously selected with a cutoff of log2 FC and Student asymptotic *p* -value were filtered from the entire list (Fig 4A). Four modules, designated green, yellow, red, and brown, were selected for further analysis, and GO term and KEGG pathway analyses were conducted. The green and yellow modules were positively associated with the SR and SR+Rego group, respectively, while the red and brown modules were negatively associated with the SR and SR+Rego group. The log2 FC values for the green and red modules were positive, indicating upregulation of gene expression in these modules, while the log2 FC values for the yellow and brown modules were negative, indicating downregulation of gene expression in these modules (Fig 4B).

Co-expression network and functional analysis of each module were subsequently conducted. The co-expression network shows 5 selected DElncRNAs and their highly correlated 10 DEmRNAs. In the green module, GO analysis revealed that the BP categories were mainly associated with angiogenesis, cell adhesion, and lipid bioprocess (Fig 4C). In the yellow module, GO analysis highlighted terms related to nucleosome assembly and epigenetic regulation of gene expression (Fig 4D). The red module has no DElncRNAs and low number of DEmRNAs compared to the other modules; its GO terms were related to synaptic membrane adhesion and cell communication (Fig 4E). In the brown module, GO analysis highlighted terms related to complement activation, apoptotic cell clearance, and BMP signaling (Fig 4F). The DEmRNAs/DElncRNAs of WGCNA are listed in S9 Table.

### Validation of DElncRNAs and DEmRNAs

**Fig 5:**
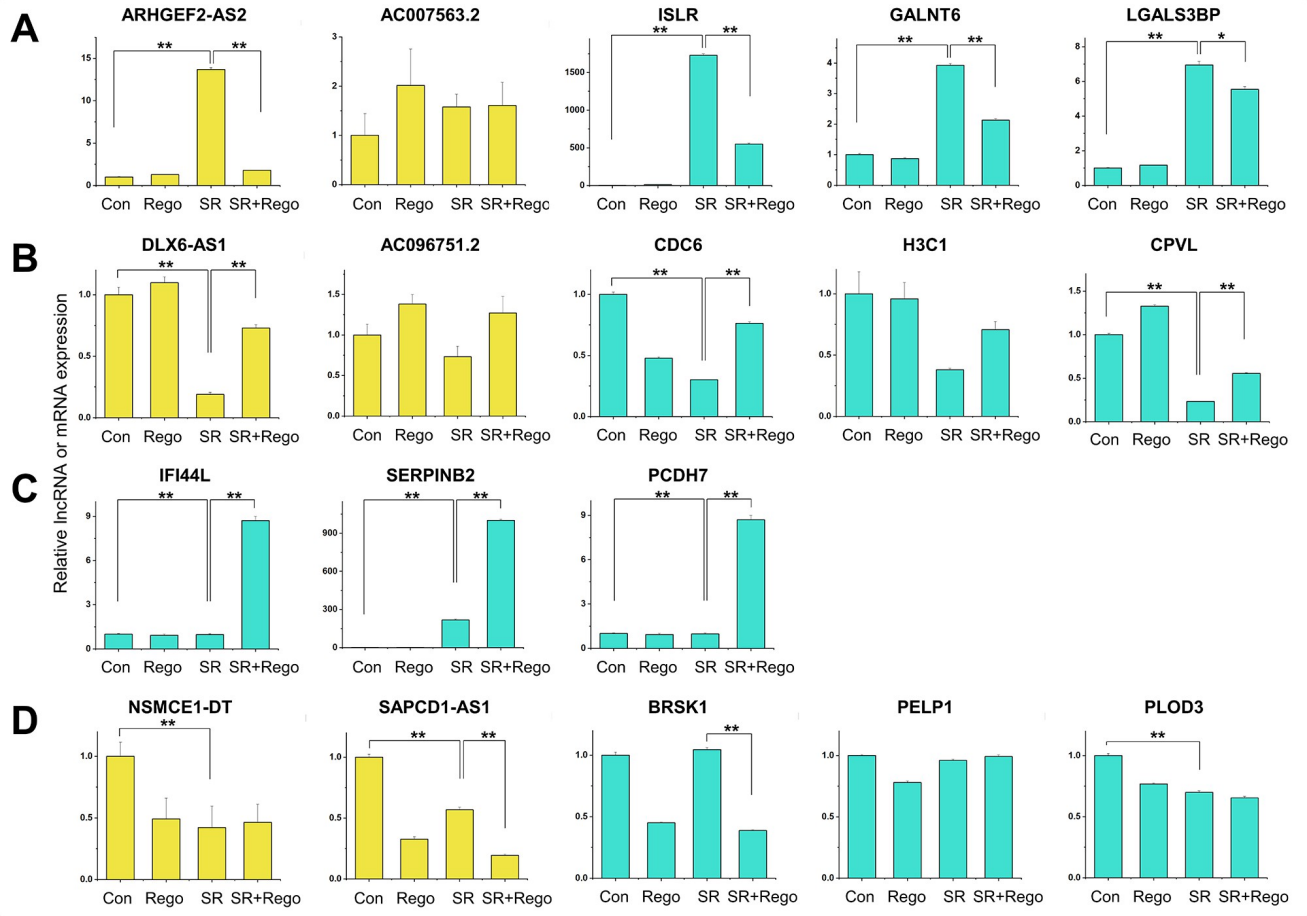
Validation of selected DEmRNAs and DElncRNAs in WGCNA analysis. Confirmation of DEmRNAs and DElncRNAs selected from four modules, MEgreen (A), MEyellow (B), MEred (C), and MEbrown (D), was evaluated by quantitative real-time PCR (qRT-PCR). Yellow bars indicate DElncRNAs, and turquoise bars indicate DEmRNAs. RNA levels were normalized to GAPDH and RNU6-1 (U6) transcript levels. The data represent three independent experiments, and the values are the mean ± SEM of triplicate experiments (*p ≤ 0.05 and **p ≤ 0.01).

In order to validate the four modules that were identified by WGCNA analysis, the expression levels of the DElncRNAs and their highly correlated DEmRNAs were measured by qRT-PCR (Fig 5). Fig 5A, which refers to the green module, shows that the Rego treatment of SR cells (SR + Rego) partially reversed the increases that occurred in the SR versus the Con cells (1 of 2 tested lncRNAs, 2 of 3 tested mRNAs). Within the yellow module (Fig 5B), the SR cells mostly exhibited decreases compared to Con, which were partially or fully reversed by Rego (1 of 2 lncRNAs, 2 of 3 mRNAs). Similar to the yellow module, the red module (Fig 5C) revealed increases in SR+Rego versus SR cells (3 of 3 mRNAs), but unlike in the yellow module, these increases were not reversals of SR vs. Con changes. In the brown module (Fig 5D), there were significant decreases in SR vs. Con cells (2 of 2 lncRNAs, 1 of 3 mRNAs), similar to the yellow module, but unlike in the yellow module, these decreases were not neutralized by Rego. Rather, where the brown module showed a difference of SR+Rego vs SR, it was a reduction in SR+Rego, similar to the green module.

### Comparative analysis of regorafenib-treated HCC cells and SR cells

**Fig 6:**
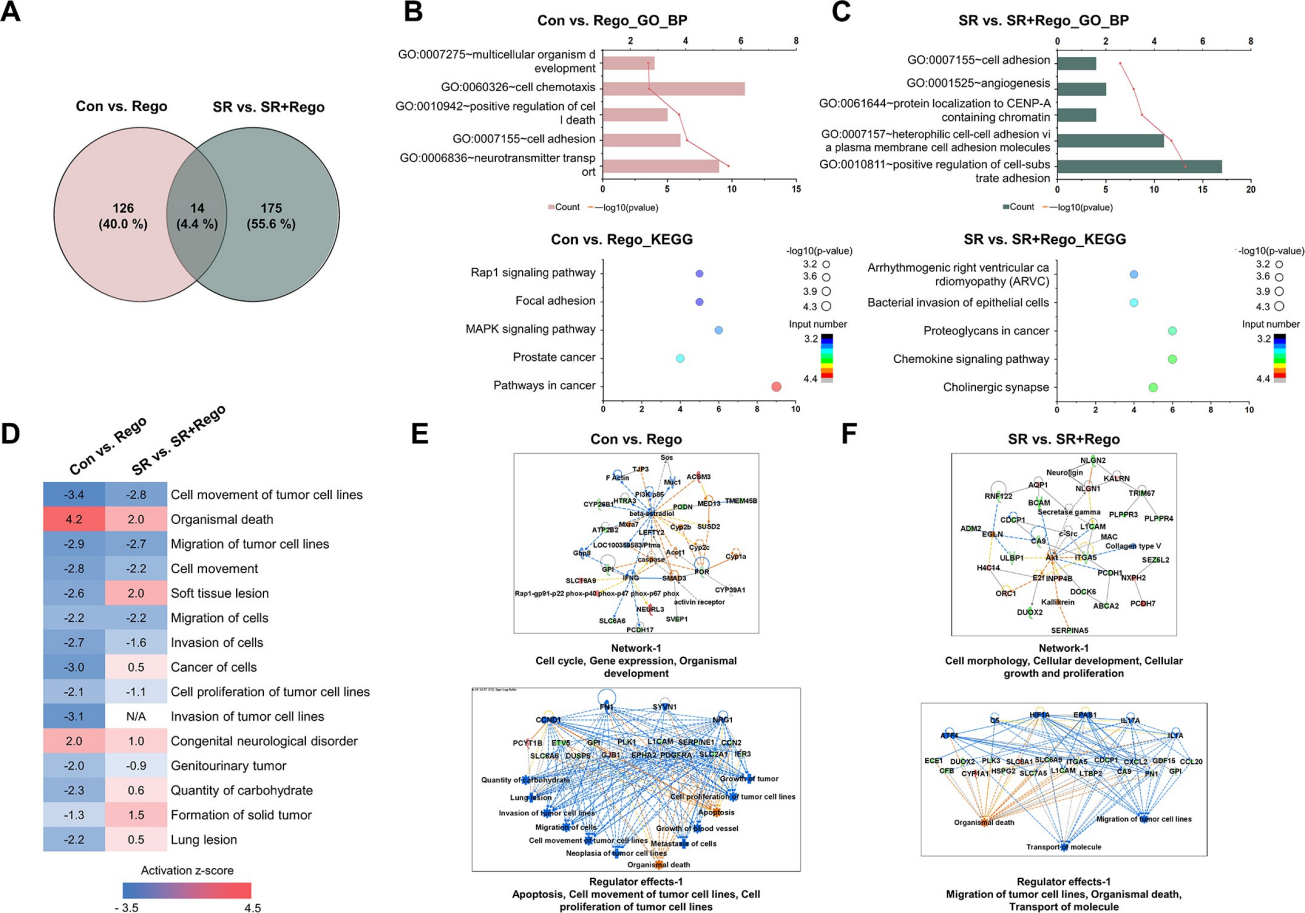
Comparative analysis of regorafenib-treated HCC cells and SR cells. The Venn diagrams (A) show the numbers of DEmRNAs in regorafenib-treated HCC cells and SR cells. GO BP terms (upper panel) and KEGG pathways (bottom panel) enriched in genes in regorafenib-treated HCC cells (B) and regorafenib-treated SR cells (C). Biofunctional analysis of alternated gene datasets (D) in Con vs. Rego and SR vs. SR+Rego using IPA software. Gene networks of Con vs. Rego (E) or SR vs. SR+Rego (F) cells by IPA software.

The above-described acute regorafenib treatment (24 hours, 5 μM) of the SR cells was finally compared with the parallel regorafenib treatment of the normal HCC cells. The results showed that different genes were regulated in each dataset, with few overlaps (Fig 6A). The Con vs. Rego dataset revealed that the BP category was mainly associated with multicellular organism development and cell chemotaxis (Fig 6B), while the SR vs. SR+Rego dataset highlighted terms related to cell adhesion and angiogenesis (Fig 6C). Biofunctional analysis of the genes derived from the Con vs. Rego and SR vs. SR+Rego datasets was conducted using Ingenuity Pathway Analysis (IPA, QIAGEN Redwood City, www.qiagen.com/ingenuity) software (Fig 6D). The results of biofunctional analysis revealed that the genes activated in both datasets were primarily enriched with terms related to cell movement, organismal death, and invasion. Notably, these functions were more prominently affected in the Rego than SR+Rego cells. Specifically, functions such as movement and invasion were more significantly reduced in the Rego group, while organismal death was substantially increased. Additionally, the network of genes and associated functions were identified, representing the interacting genes within each dataset. The network of genes is illustrated in Fig 6E, while the regulatory effects are depicted in Fig 6F. Associations of cell cycle with gene expression or genes related to apoptosis and organism mission were consistent with the GO analysis results.

## Discussion

In this study, we aimed to understand how regorafenib treatment affects the gene expression profiles of HCCs in the context of SR. By examining DEmRNAs and DElncRNAs, we gained insights into the molecular changes and potential mechanisms underlying the response to regorafenib in SR cells.

To accomplish this objective, we generated SR cells and confirmed their reduced sensitivity and reduced responsiveness to sorafenib through cell viability, apoptosis, and colony formation assays. It is known that the SR of HCC involves various signaling pathways [23], amongst them the activation of the PI3K/Akt pathway, which facilitates their survival, impedes apoptosis, and boosts growth [24, 25]. The present study confirms the upregulation of PI3K-Akt signaling in SR cells (S4 Fig and Fig 3). Typically, upregulation of PI3K-Akt signaling is associated with increased cell invasion or migration [26]. However, our GO results did not support such a relationship. This reveals an important limitation of regorafenib that needs to be addressed in the future, but also indicates that its anti-tumor activity in the context of SR involves alternative mechanisms.

One such alternative mechanism could be TGF-beta signaling. This pathway generally promotes EMT and was shown to be active in HCC, where it promotes tumor growth, invasion, and metastasis and correlates with poor prognosis [27–29]. Significantly, sorafenib exacerbates the problem by increasing TGF-β expression and promoting EMT [30]. In contrast, we found that regorafenib reverses the elevated TGF-beta signaling in SR cells (Fig 3), at least in the acute treatment period of 24 hours studied here. The importance of this pathway is in line with the major role of EMT in regorafenib resistance [31].

Another mechanism whereby regorafenib exerts its anti-tumor activity in the SR context could be epigenetic. Through GO and gene expression pattern analyses, we observed the upregulation of genes involved in nucleosome assembly and epigenetic gene expression. These findings were supported by the nucleosome assembly, epigenetic regulation of gene expression, and protein localization to CENP-A containing chromatin, as seen in the yellow module of the WGCNA analysis (Fig 4). These results imply that regorafenib induces chromatin remodeling and thereby reprograms gene expression. However, whether these alterations are predominantly desirable or not remains unclear. Recent reports highlight the involvement of epigenetic modulators in HCC development and progression [32], and various epigenetic alterations, including chromatin structure remodeling, DNA methylation, post-translational histone modifications, and changes in non-coding RNA levels, contribute to the remodeling of the tumor microenvironment in HCC patients [33]. Furthermore, targeting specific epigenetic regulators, such as EZH2 and DNMT1, can enhance the response to immunotherapy in HCC patients [34]. On the other hand, it is known that chemotherapeutic agents can induce resistance via epigenetic reprogramming [35, 36], which—in turn—can be mitigated by epigenetic modifiers [37]. Thus, the relationship between regorafenib and epigenetic regulation and its potential clinical use requires further investigation.

The present study focused on the (acute) effects of regorafenib on the DElncRNA and DEmRNA expression patterns of (chronically) sorafenib-treated HCC cells. We observed the up- or down-regulation of genes associated with multiple molecular functions and metabolic processes. Previously, Wu et al. reported differentially expressed lncRNAs in Sorafenib-resistant Huh7 and HepG2 cells, influencing binding, catalytic activity, and biological regulation of metabolic processes [38]. Our study did not yield identical results. While discrepancies may arise from differences in the conditions used to establish SR, as well as variations in the processing of the RNA-seq dataset, the consistent findings across studies suggest that several signaling pathways and metabolic processes undergo alterations in SR cells.

Previous studies have identified a complex network of lncRNAs and mRNAs that contribute to sorafenib resistance in liver cancer [39, 40]. Various lncRNAs were shown to regulate sorafenib resistance through diverse pathways, including AKT activation, apoptosis, and EMT [41]. Ye et al. applied WGCNA to RNA-Seq data from HCC cell lines and discovered 13 gene modules involved in HCC initiation, prognosis, and drug resistance, thus providing potentially useful information for HCC diagnosis and treatment [42].

A further emphasis of the current study was to explore potential regulatory mechanisms of lncRNAs in the regorafenib-treated SR HCC cells. Here, we used WGCNA to analyze the interactions between DElncRNAs and DEmRNAs of SR cells under acute exposure to regorafenib. This approach enhances our understanding of the involvement of these molecular networks in cancer progression and treatment. Such analyses play a pivotal role in identifying crucial lncRNAs associated with the development and progression of cancer, shedding light on their functional roles in these processes. We identified several modules of co-expressed DElncRNAs and DEmRNAs (Fig 4), four of which showed significant associations with key cellular processes such as proliferation, adhesion, nucleosome assembly, and apoptosis. Notable lncRNAs of the green module (increased in SR, then decreased under regorafenib) include ARHGEF2-AS2, a survival predictor in clear cell renal cell carcinoma (ccRCC) [43], and AC007563.2, AC013451.1, LINC00607, and LINC01583, which are potentially involved in angiogenesis and cell adhesion [44–47]. Notable lncRNAs of the yellow module (decreased in SR, then increased after regorafenib) include the oncogenic molecules CHRM3-AS2 [48] and DLX6-AS1 [49] as well as AL137127.1, AC020978.3 and LINC01242, which are thought to promote chromatin remodeling in HCC [50, 51]. The red module (increased in SR, further increased with regorafenib) did not contain any DElncRNAs, and the DEmRNAs in this module have been reported to act as tumor suppressors [52–54]. Finally, the brown module (decreased in SR, further decreased with regorafenib) contained NSMCE1-DT, a lipid metabolism-related prognostic lncRNAs that is overexpressed in colorectal cancer and negatively associated with survival [55]. The expression patterns of DElncRNAs and DEmRNAs in the brown module were not entirely consistent, however; it was more a tendency to decrease following regorafenib treatment. SAPCD1-AS1, AC012615.1, AC107959.3, and PCED1B-AS1 may regulate various cellular responses.

## Conclusions

Using a cell culture model, we characterized the acute transcriptional response of SR HCC cells to the second-line chemotherapeutic, regorafenib. In this context, we identified regorafenib-responsive signaling pathways and DElncRNAs/DEmRNAs co-expression modules with regulatory potential. From a clinical perspective, some effects appear beneficial but others are reason for concern. Future work will need to study the long-term exposure of SR HCC cells to regorafenib.

## Supporting information

S1 Fig GO and KEGG pathway analysis of the DEmRNAs in Rego cells. Shown are the GO term with up- (A) and downregulated (C) genes and KEGG pathway enrichment with up- (B) and down-regulated (D) genes analyses of the Rego cells. In the GO term analyses, the numbers of genes and *p-*values are displayed for the top 5 GO terms in biological process (BP; upper panel and blue column), cellular component (CC; middle panel and yellow column), and molecular functions (MF; bottom panel and pink column). The column is the count value indicating the number of genes enriched in the GO term, and the red line is the -log_10_ (*p-*value) value. In the KEGG pathway enrichment analyses, the *x*-axis indicates the count value, and the size of the bubble indicates the -log_10_ (*p-*value).
(TIFF)

S2 Fig GO and KEGG pathway analyses of the DEmRNAs in SR cells. Shown are the GO term with up- (A) and downregulated (C) genes and KEGG pathway enrichment with up- (B) and down-regulated (D) genes analyses of the SR cells. In the GO term analyses, the numbers of genes and p-values are displayed for the top 5 GO terms in BP (upper panel and blue column), CC (middle panel and yellow column), and MF (bottom panel and pink column). The column is the count value indicating the number of genes enriched in the GO term, and the red line is the -log_10_ (*p*-value) value. In the KEGG pathway enrichment analyses, the x-axis indicates the count value, and the size of the bubble indicates the -log_10_ (*p*-value).
(TIFF)

S3 Fig GO and KEGG pathway analyses of the DEmRNAs in SR+Rego cells. Shown are the GO term with up- (A) and downregulated (C) genes and KEGG pathway enrichment with up- (B) and down-regulated (D) genes analyses of the SR+Rego cells. In the GO term analyses, the numbers of genes and p-values are displayed for the top 5 GO terms in BP (upper panel and blue column), CC (middle panel and yellow column), and MF (bottom panel and pink column). The column is the count value indicating the number of genes enriched in the GO term, and the red line is the -log_10_ (*p*-value) value. In the KEGG pathway enrichment analyses, the x-axis indicates the count value, and the size of the bubble indicates the -log_10_ (*p*-value).
(TIFF)

S4 Fig GO and KEGG pathway analyses of the commonly expressed DEmRNAs in SR and SR+Rego cells. (A, B) GO term analysis of the commonly associated BPs with (A) upregulated and (B) downregulated genes. The top 10 enriched GO terms in BPs are shown, with the number of genes and *p*-values displayed. The column represents the count value indicating the number of genes enriched in the GO term, and the black line represents the–log_10_ (*p*-value) value. The red bar and solid black line indicate the SR cells, while the cyan bar and dotted black line indicate the SR+Rego cells. (C, D) KEGG pathway analysis of the commonly associated pathways with (C) upregulated and (D) downregulated genes. The size of the bubble represents the count value, and the color represents the *p*-value. Circle and triangle symbols indicate SR and SR+Rego cells, respectively.
(TIFF)

S5 Fig GO and KEGG pathway analyses of each group of DEmRNAs. (A–D) GO term analysis of (A) upregulated genes in SR cells, (B) downregulated genes in SR cells; (C) upregulated genes in SR+Rego cells; (D) downregulated genes in SR+Rego cells. The numbers of genes and *p*-values are displayed for the top 10 GO terms in BP category. The column represents the count value indicating the number of genes enriched in the GO term, and the black line represents the -log_10_ (*p*-value) value. (E, F) KEGG pathway enrichment analysis of (E) upregulated and (F) downregulated genes in SR cells. (G) KEGG pathway enrichment analysis of upregulated genes in SR+Rego cells. The size of the bubble represents the count value, and the color represents the–log_10_ (*p*-value).
(TIFF)

S6 Fig Functional annotation of DElncRNAs in SR cells. GREAT computes all GO term enrichment for genes upstream and downstream of TSS based on the genomic regions of DElncRNAs commonly expressed in SR cells. Distance (kb) to the nearest transcriptional start site (TSS) and Absolute distance to DElncRNAs and TSS sites of up- (A) and downregulated (B) lncRNAs in SR cells. Functional annotation analysis of up- (C) and downregulated (D) DElncRNAs using GREAT shows for the top 5 GO terms in BP (top panel), CC (middle panel), and MF (bottom panel).
(TIFF)

S7 Fig Functional annotation of DElncRNAs in SR+Rego cells. GREAT computes all GO term enrichment for genes upstream and downstream of TSS based on the genomic regions of DElncRNAs commonly expressed in SR+Rego cells. Distance (kb) to the nearest transcriptional start site (TSS) and Absolute distance to DElncRNAs and TSS sites of up- (A) and downregulated(B) lncRNAs in SR+Rego cells. Functional annotation analysis of up- (C) and downregulated (D) DElncRNAs using GREAT shows for the top 5 GO terms in BP (top panel), CC (middle panel), and MF (bottom panel).
(TIFF)

S1 Table List of primers used for qRT-PCR. (CSV)

S2 Table All significant up- and downregulated DEmRNAs in Rego vs. Con cells. (CSV)

S3 Table All significant up- and downregulated DElncRNAs in Rego vs. Con cells. (CSV)

S4 Table All significant up- and downregulated DEmRNAs in SR vs. Con cells. (CSV)

S5 Table All significant up- and downregulated DElncRNAs in SR vs. Con cells. (CSV)

S6 Table All significant up- and downregulated DEmRNAs in SR+Rego vs. Con cells. (CSV)

S7 Table All significant up- and downregulated DElncRNAs in SR+Rego vs. Con cells. (CSV)

S8 Table All significant up- and downregulated DEmRNAs and DElncRNAs in gene expression pattern analysis. (CSV)

S9 Table All significant up- and downregulated DEmRNAs and DElncRNAs in WGNCA. (CSV)

## Abbreviations

AKT: Protein kinase B signaling
BP: Biological process
CC: Cellular Component
DAVID: Database for Annotation, Visualization, and Integrated Discovery
DElncRNAs: Differentially expressed lncRNAs
DEmRNAs: Differentially expressed mRNAs
GO: Gene Ontology
HCC: Hepatocellular carcinoma
IPA: Ingenuity Pathway Analysis
KEGG: Kyoto Encyclopedia of Genes and Genomes
KOBAS: KEGG Orthology Based Annotation System
LncRNA: Long non-coding RNA
MF: Molecular Functions
qRT-PCR: Quantitative reverse transcription PCR
Rego: Regorafenib
RNA-seq: RNA sequencing
SR: Sorafenib resistant
SR+Rego: Rego-treated SR
WGCNA: Weighted gene co-expression network analysis

## References

[1] Llovet JM, Kelley RK, Villanueva A, Singal AG, Pikarsky E, Roayaie S, et al. Hepatocellular carcinoma. Nat Rev Dis Primers. 2021;7(1):6. doi: 10.1038/s41572-020-00240-3 33479224 PMC13537433

[2] Yang C, Zhang H, Zhang L, Zhu AX, Bernards R, Qin W, et al. Evolving therapeutic landscape of advanced hepatocellular carcinoma. Nat Rev Gastroenterol Hepatol. 2022. doi: 10.1038/s41575-022-00704-9 36369487

[3] Tang W, Chen Z, Zhang W, Cheng Y, Zhang B, Wu F, et al. The mechanisms of sorafenib resistance in hepatocellular carcinoma: theoretical basis and therapeutic aspects. Signal Transduct Target Ther. 2020;5(1):87. doi: 10.1038/s41392-020-0187-x 32532960 PMC7292831

[4] Huang A, Yang XR, Chung WY, Dennison AR, Zhou J. Targeted therapy for hepatocellular carcinoma. Signal Transduct Target Ther. 2020;5(1):146. doi: 10.1038/s41392-020-00264-x 32782275 PMC7419547

[5] Bruix J, Qin S, Merle P, Granito A, Huang YH, Bodoky G, et al. Regorafenib for patients with hepatocellular carcinoma who progressed on sorafenib treatment (RESORCE): a randomised, double-blind, placebo-controlled, phase 3 trial. Lancet. 2017;389(10064):56–66. doi: 10.1016/S0140-6736(16)32453-9 27932229

[6] Palazzo AF, Koonin EV. Functional Long Non-coding RNAs Evolve from Junk Transcripts. Cell. 2020;183(5):1151–61. doi: 10.1016/j.cell.2020.09.047 33068526

[7] Statello L, Guo CJ, Chen LL, Huarte M. Gene regulation by long non-coding RNAs and its biological functions. Nat Rev Mol Cell Biol. 2021;22(2):96–118. doi: 10.1038/s41580-020-00315-9 33353982 PMC7754182

[8] Iaccarino I, Klapper W. LncRNA as Cancer Biomarkers. Methods Mol Biol. 2021;2348:27–41. doi: 10.1007/978-1-0716-1581-2_2 34160797

[9] Eptaminitaki GC, Stellas D, Bonavida B, Baritaki S. Long non-coding RNAs (lncRNAs) signaling in cancer chemoresistance: From prediction to druggability. Drug Resist Updat. 2022;65:100866. doi: 10.1016/j.drup.2022.100866 36198236

[10] Chen B, Dragomir MP, Yang C, Li Q, Horst D, Calin GA. Targeting non-coding RNAs to overcome cancer therapy resistance. Signal Transduct Target Ther. 2022;7(1):121. doi: 10.1038/s41392-022-00975-3 35418578 PMC9008121

[11] Fan L, Huang X, Chen J, Zhang K, Gu YH, Sun J, et al. Long Noncoding RNA MALAT1 Contributes to Sorafenib Resistance by Targeting miR-140-5p/Aurora-A Signaling in Hepatocellular Carcinoma. Mol Cancer Ther. 2020;19(5):1197–209. doi: 10.1158/1535-7163.MCT-19-0203 32220970

[12] Chen S, Xia X. Long noncoding RNA NEAT1 suppresses sorafenib sensitivity of hepatocellular carcinoma cells via regulating miR-335-c-Met. J Cell Physiol. 2019;234(9):14999–5009. doi: 10.1002/jcp.27567 30937906

[13] Li W, Dong X, He C, Tan G, Li Z, Zhai B, et al. Correction to: LncRNA SNHG1 contributes to sorafenib resistance by activating the Akt pathway and is positively regulated by miR-21 in hepatocellular carcinoma cells. J Exp Clin Cancer Res. 2021;40(1):377. doi: 10.1186/s13046-021-02183-3 34852817 PMC8638182

[14] Baek M, Chai JC, Choi HI, Yoo E, Binas B, Lee YS, et al. Comprehensive transcriptome profiling of BET inhibitor-treated HepG2 cells. PLoS One. 2022;17(4):e0266966. doi: 10.1371/journal.pone.0266966 35486664 PMC9053788

[15] Baek M, Chai JC, Choi HI, Yoo E, Binas B, Lee YS, et al. Analysis of differentially expressed long non-coding RNAs in LPS-induced human HMC3 microglial cells. BMC Genomics. 2022;23(1):853. doi: 10.1186/s12864-022-09083-6 36575377 PMC9795738

[16] Bolger AM, Lohse M, Usadel B. Trimmomatic: a flexible trimmer for Illumina sequence data. Bioinformatics. 2014;30(15):2114–20. doi: 10.1093/bioinformatics/btu170 24695404 PMC4103590

[17] Dobin A, Davis CA, Schlesinger F, Drenkow J, Zaleski C, Jha S, et al. STAR: ultrafast universal RNA-seq aligner. Bioinformatics. 2013;29(1):15–21. doi: 10.1093/bioinformatics/bts635 23104886 PMC3530905

[18] Love MI, Huber W, Anders S. Moderated estimation of fold change and dispersion for RNA-seq data with DESeq2. Genome Biol. 2014;15(12):550. doi: 10.1186/s13059-014-0550-8 25516281 PMC4302049

[19] Huang da W, Sherman BT, Lempicki RA. Systematic and integrative analysis of large gene lists using DAVID bioinformatics resources. Nat Protoc. 2009;4(1):44–57. doi: 10.1038/nprot.2008.211 19131956

[20] Bu D, Luo H, Huo P, Wang Z, Zhang S, He Z, et al. KOBAS-i: intelligent prioritization and exploratory visualization of biological functions for gene enrichment analysis. Nucleic Acids Res. 2021;49(W1):W317–W25. doi: 10.1093/nar/gkab447 34086934 PMC8265193

[21] Shannon P, Markiel A, Ozier O, Baliga NS, Wang JT, Ramage D, et al. Cytoscape: a software environment for integrated models of biomolecular interaction networks. Genome Res. 2003;13(11):2498–504. doi: 10.1101/gr.1239303 14597658 PMC403769

[22] Langfelder P, Horvath S. WGCNA: an R package for weighted correlation network analysis. BMC Bioinformatics. 2008;9:559. doi: 10.1186/1471-2105-9-559 19114008 PMC2631488

[23] Marin JJG, Macias RIR, Monte MJ, Romero MR, Asensio M, Sanchez-Martin A, et al. Molecular Bases of Drug Resistance in Hepatocellular Carcinoma. Cancers (Basel). 2020;12(6). doi: 10.3390/cancers12061663 32585893 PMC7352164

[24] Dong J, Zhai B, Sun W, Hu F, Cheng H, Xu J. Activation of phosphatidylinositol 3-kinase/AKT/snail signaling pathway contributes to epithelial-mesenchymal transition-induced multi-drug resistance to sorafenib in hepatocellular carcinoma cells. PLoS One. 2017;12(9):e0185088. doi: 10.1371/journal.pone.0185088 28934275 PMC5608310

[25] Huang A, Zeng P, Li Y, Lu W, Lai Y. LY294002 Is a Promising Inhibitor to Overcome Sorafenib Resistance in FLT3-ITD Mutant AML Cells by Interfering With PI3K/Akt Signaling Pathway. Front Oncol. 2021;11:782065. doi: 10.3389/fonc.2021.782065 34820336 PMC8606661

[26] Paskeh MDA, Ghadyani F, Hashemi M, Abbaspour A, Zabolian A, Javanshir S, et al. Biological impact and therapeutic perspective of targeting PI3K/Akt signaling in hepatocellular carcinoma: Promises and Challenges. Pharmacol Res. 2023;187:106553. doi: 10.1016/j.phrs.2022.106553 36400343

[27] Giannelli G, Villa E, Lahn M. Transforming growth factor-beta as a therapeutic target in hepatocellular carcinoma. Cancer Res. 2014;74(7):1890–4. 24638984 10.1158/0008-5472.CAN-14-0243

[28] Dituri F, Mancarella S, Cigliano A, Chieti A, Giannelli G. TGF-beta as Multifaceted Orchestrator in HCC Progression: Signaling, EMT, Immune Microenvironment, and Novel Therapeutic Perspectives. Semin Liver Dis. 2019;39(1):53–69. 30586675 10.1055/s-0038-1676121

[29] Garcia-Lezana T, Lopez-Canovas JL, Villanueva A. Signaling pathways in hepatocellular carcinoma. Adv Cancer Res. 2021;149:63–101. doi: 10.1016/bs.acr.2020.10.002 33579428

[30] Shrestha R, Prithviraj P, Bridle KR, Crawford DHG, Jayachandran A. Combined Inhibition of TGF-beta1-Induced EMT and PD-L1 Silencing Re-Sensitizes Hepatocellular Carcinoma to Sorafenib Treatment. J Clin Med. 2021;10(9). 10.3390/jcm10091889 PMC8123871 33925488

[31] Wang J, Zhang N, Han Q, Lu W, Wang L, Yang D, et al. Pin1 inhibition reverses the acquired resistance of human hepatocellular carcinoma cells to Regorafenib via the Gli1/Snail/E-cadherin pathway. Cancer Lett. 2019;444:82–93. doi: 10.1016/j.canlet.2018.12.010 30583078 PMC6492280

[32] Yousef MH, El-Fawal HAN, Abdelnaser A. Hepigenetics: A Review of Epigenetic Modulators and Potential Therapies in Hepatocellular Carcinoma. Biomed Res Int. 2020;2020:9593254. doi: 10.1155/2020/9593254 33299889 PMC7707949

[33] Oura K, Morishita A, Hamaya S, Fujita K, Masaki T. The Roles of Epigenetic Regulation and the Tumor Microenvironment in the Mechanism of Resistance to Systemic Therapy in Hepatocellular Carcinoma. Int J Mol Sci. 2023;24(3). doi: 10.3390/ijms24032805 36769116 PMC9917861

[34] Hong YK, Li Y, Pandit H, Li S, Pulliam Z, Zheng Q, et al. Epigenetic modulation enhances immunotherapy for hepatocellular carcinoma. Cell Immunol. 2019;336:66–74. doi: 10.1016/j.cellimm.2018.12.010 30626493

[35] Li GH, Qu Q, Qi TT, Teng XQ, Zhu HH, Wang JJ, et al. Super-enhancers: a new frontier for epigenetic modifiers in cancer chemoresistance. J Exp Clin Cancer Res. 2021;40(1):174. doi: 10.1186/s13046-021-01974-y 34011395 PMC8132395

[36] Quagliano A, Gopalakrishnapillai A, Barwe SP. Understanding the Mechanisms by Which Epigenetic Modifiers Avert Therapy Resistance in Cancer. Front Oncol. 2020;10:992. doi: 10.3389/fonc.2020.00992 32670880 PMC7326773

[37] Jin N, George TL, Otterson GA, Verschraegen C, Wen H, Carbone D, et al. Advances in epigenetic therapeutics with focus on solid tumors. Clin Epigenetics. 2021;13(1):83. doi: 10.1186/s13148-021-01069-7 33879235 PMC8056722

[38] Wu M, Shen X, Tang Y, Zhou C, Li H, Luo X. Identification and validation of potential key long noncoding RNAs in sorafenib-resistant hepatocellular carcinoma cells. PeerJ. 2020;8:e8624. doi: 10.7717/peerj.8624 32149026 PMC7049252

[39] Wong CM, Tsang FH, Ng IO. Non-coding RNAs in hepatocellular carcinoma: molecular functions and pathological implications. Nat Rev Gastroenterol Hepatol. 2018;15(3):137–51. doi: 10.1038/nrgastro.2017.169 29317776

[40] Lim LJ, Wong SYS, Huang F, Lim S, Chong SS, Ooi LL, et al. Roles and Regulation of Long Noncoding RNAs in Hepatocellular Carcinoma. Cancer Res. 2019;79(20):5131–9. doi: 10.1158/0008-5472.CAN-19-0255 31337653

[41] Huang Z, Zhou JK, Peng Y, He W, Huang C. The role of long noncoding RNAs in hepatocellular carcinoma. Mol Cancer. 2020;19(1):77. doi: 10.1186/s12943-020-01188-4 32295598 PMC7161154

[42] Ye H, Sun M, Huang S, Xu F, Wang J, Liu H, et al. Gene Network Analysis of Hepatocellular Carcinoma Identifies Modules Associated with Disease Progression, Survival, and Chemo Drug Resistance. Int J Gen Med. 2021;14:9333–47. doi: 10.2147/IJGM.S336729 34898998 PMC8654693

[43] Bai Z, Zhao Y, Yang X, Wang L, Yin X, Chen Y, et al. A Novel Prognostic Ferroptosis-Related Long Noncoding RNA Signature in Clear Cell Renal Cell Carcinoma. J Oncol. 2022;2022:6304824. doi: 10.1155/2022/6304824 35242188 PMC8888116

[44] Ghafouri-Fard S, Asadi M, Sohrabi B, Arsang-Jang S, Mehravaran E, Taheri M, et al. Down-regulation of a panel of immune-related lncRNAs in breast cancer. Pathol Res Pract. 2021;224:153534. doi: 10.1016/j.prp.2021.153534 34175685

[45] Qi F, Du X, Zhao Z, Zhang D, Huang M, Bai Y, et al. Tumor Mutation Burden-Associated LINC00638/miR-4732-3p/ULBP1 Axis Promotes Immune Escape via PD-L1 in Hepatocellular Carcinoma. Front Oncol. 2021;11:729340. doi: 10.3389/fonc.2021.729340 34568062 PMC8456090

[46] Vishnubalaji R, Alajez NM. Epigenetic regulation of triple negative breast cancer (TNBC) by TGF-beta signaling. Sci Rep. 2021;11(1):15410. 34326372 10.1038/s41598-021-94514-9 PMC8322425

[47] Zhao J, Xu J, Shang AQ, Zhang R. A Six-LncRNA Expression Signature Associated with Prognosis of Colorectal Cancer Patients. Cell Physiol Biochem. 2018;50(5):1882–90. doi: 10.1159/000494868 30396175

[48] Wang D, Chen Q, Liu J, Liao Y, Jiang Q. Silencing of lncRNA CHRM3-AS2 Expression Exerts Anti-Tumour Effects Against Glioma via Targeting microRNA-370-5p/KLF4. Front Oncol. 2022;12:856381. doi: 10.3389/fonc.2022.856381 35359381 PMC8962832

[49] Ghafouri-Fard S, Najafi S, Hussen BM, Ganjo AR, Taheri M, Samadian M. DLX6-AS1: A Long Non-coding RNA With Oncogenic Features. Front Cell Dev Biol. 2022;10:746443. doi: 10.3389/fcell.2022.746443 35281110 PMC8916230

[50] Li X, Yang L, Wang W, Rao X, Lai Y. Constructing a prognostic immune-related lncRNA model for colon cancer. Medicine (Baltimore). 2022;101(38):e30447. doi: 10.1097/MD.0000000000030447 36197160 PMC9509170

[51] Liu C, Wu S, Lai L, Liu J, Guo Z, Ye Z, et al. Comprehensive analysis of cuproptosis-related lncRNAs in immune infiltration and prognosis in hepatocellular carcinoma. BMC Bioinformatics. 2023;24(1):4. doi: 10.1186/s12859-022-05091-1 36597032 PMC9811804

[52] Chen HF, Ma RR, He JY, Zhang H, Liu XL, Guo XY, et al. Protocadherin 7 inhibits cell migration and invasion through E-cadherin in gastric cancer. Tumour Biol. 2017;39(4):1010428317697551. doi: 10.1177/1010428317697551 28381163

[53] Chen Z, Wei Y, Zheng Y, Zhu H, Teng Q, Lin X, et al. SERPINB2, an Early Responsive Gene to Epigallocatechin Gallate, Inhibits Migration and Promotes Apoptosis in Esophageal Cancer Cells. Cells. 2022;11(23). doi: 10.3390/cells11233852 36497110 PMC9738437

[54] Huang WC, Tung SL, Chen YL, Chen PM, Chu PY. IFI44L is a novel tumor suppressor in human hepatocellular carcinoma affecting cancer stemness, metastasis, and drug resistance via regulating met/Src signaling pathway. BMC Cancer. 2018;18(1):609. doi: 10.1186/s12885-018-4529-9 29848298 PMC5977745

[55] Lin Y, Xiao Y, Liu S, Hong L, Shao L, Wu J. Role of a lipid metabolism-related lncRNA signature in risk stratification and immune microenvironment for colon cancer. BMC Med Genomics. 2022;15(1):221. doi: 10.1186/s12920-022-01369-8 36280825 PMC9590147

