# Supplementary figures and images for: Identification of differentially expressed mRNA/lncRNA modules in acutely regorafenib-treated sorafenib-resistant Huh7 hepatocellular carcinoma cells
Source: PLoS One. 2024 Apr 11;19(4):e0301663. doi: 10.1371/journal.pone.0301663 (PMC11008899; doi:10.1371/journal.pone.0301663)

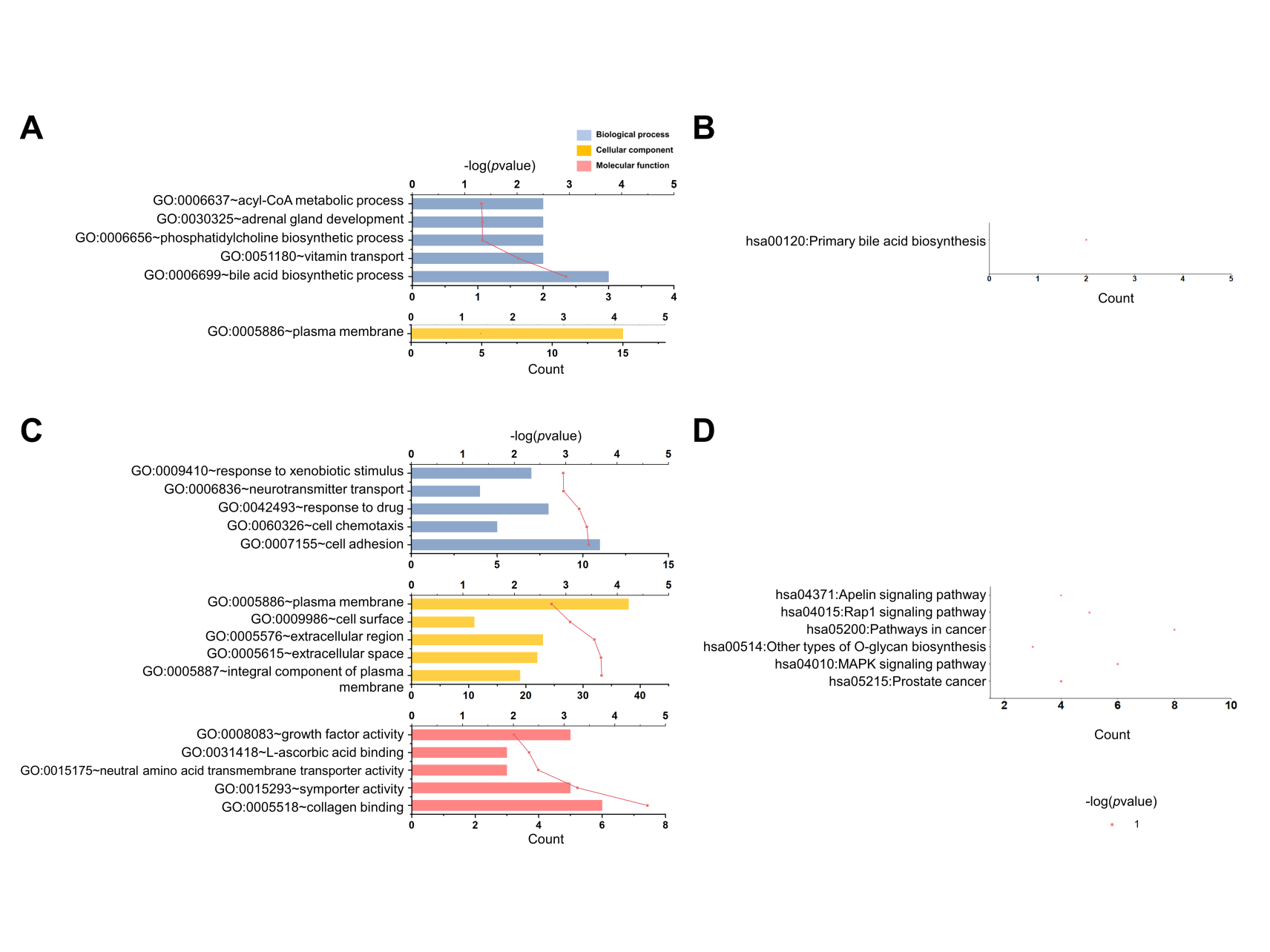

Supplement: S1 Fig — Shown are the GO term with up- (A) and downregulated (C) genes and KEGG pathway enrichment with up- (B) and down-regulated (D) genes analyses of the Rego cells. In the GO term analyses, the numbers of genes and p-values are displayed for the top 5 GO terms in biological process (BP; upper panel and blue column), cellular component (CC; middle panel and yellow column), and molecular functions (MF; bottom panel and pink column). The column is the count value indicating the number of genes enriched in the GO term, and the red line is the -log10 (p-value) value. In the KEGG pathway enrichment analyses, the x-axis indicates the count value, and the size of the bubble indicates the -log10 (p-value). (TIFF) [file pone.0301663.s001.tiff]

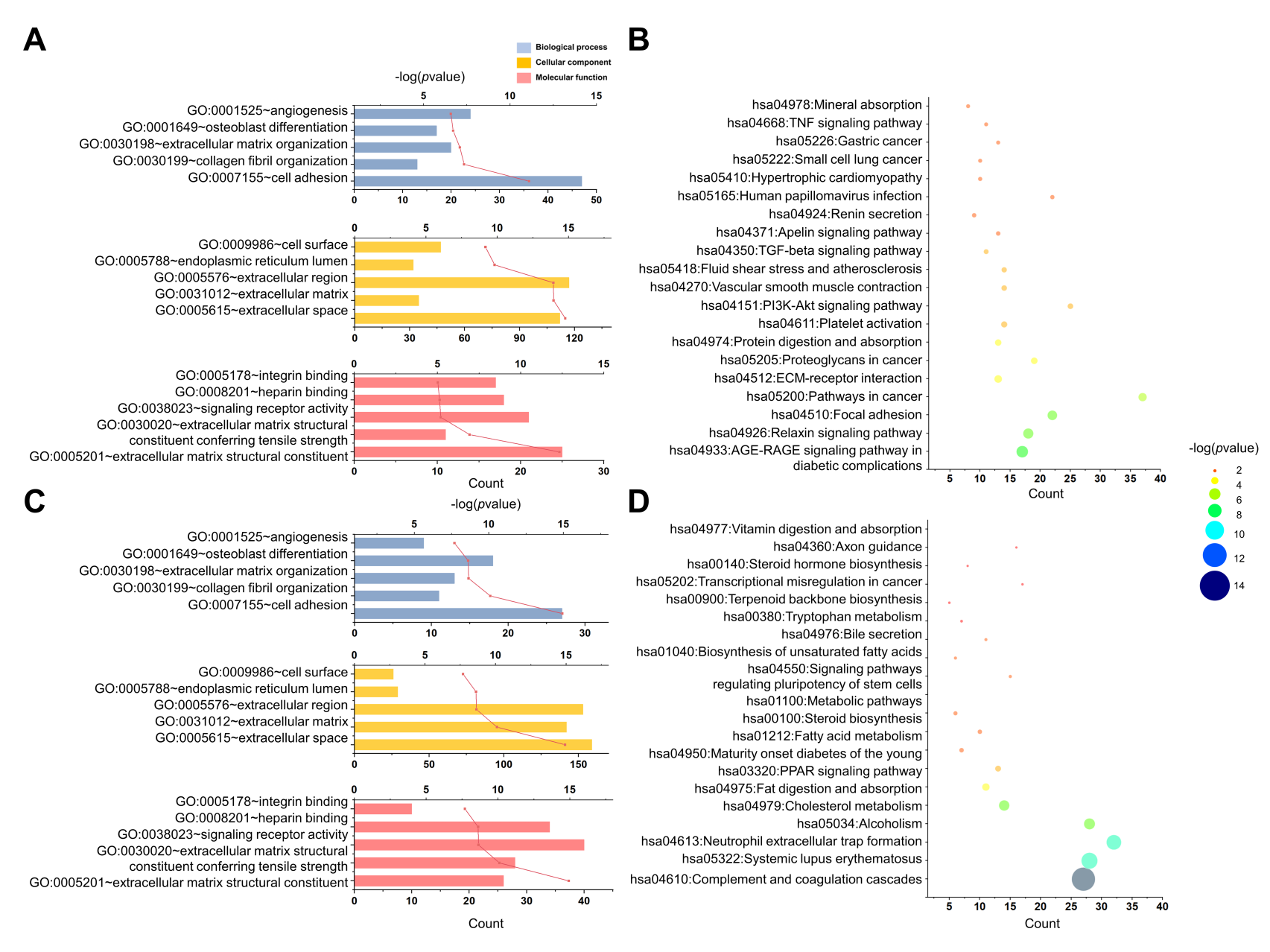

Supplement: S2 Fig — Shown are the GO term with up- (A) and downregulated (C) genes and KEGG pathway enrichment with up- (B) and down-regulated (D) genes analyses of the SR cells. In the GO term analyses, the numbers of genes and p-values are displayed for the top 5 GO terms in BP (upper panel and blue column), CC (middle panel and yellow column), and MF (bottom panel and pink column). The column is the count value indicating the number of genes enriched in the GO term, and the red line is the -log10 (p-value) value. In the KEGG pathway enrichment analyses, the x-axis indicates the count value, and the size of the bubble indicates the -log10 (p-value). (TIFF) [file pone.0301663.s002.tiff]

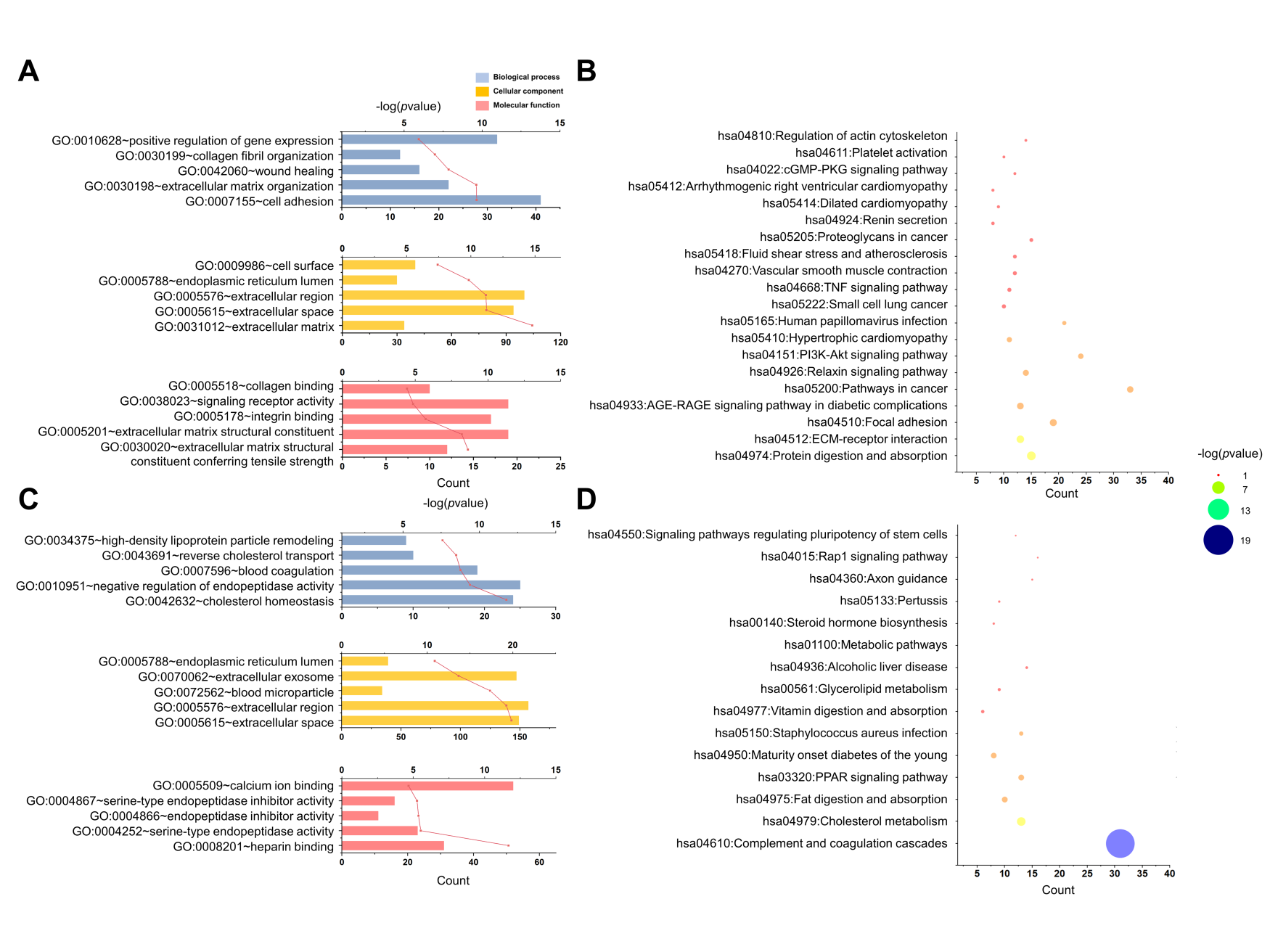

Supplement: S3 Fig — Shown are the GO term with up- (A) and downregulated (C) genes and KEGG pathway enrichment with up- (B) and down-regulated (D) genes analyses of the SR+Rego cells. In the GO term analyses, the numbers of genes and p-values are displayed for the top 5 GO terms in BP (upper panel and blue column), CC (middle panel and yellow column), and MF (bottom panel and pink column). The column is the count value indicating the number of genes enriched in the GO term, and the red line is the -log10 (p-value) value. In the KEGG pathway enrichment analyses, the x-axis indicates the count value, and the size of the bubble indicates the -log10 (p-value). (TIFF) [file pone.0301663.s003.tiff]

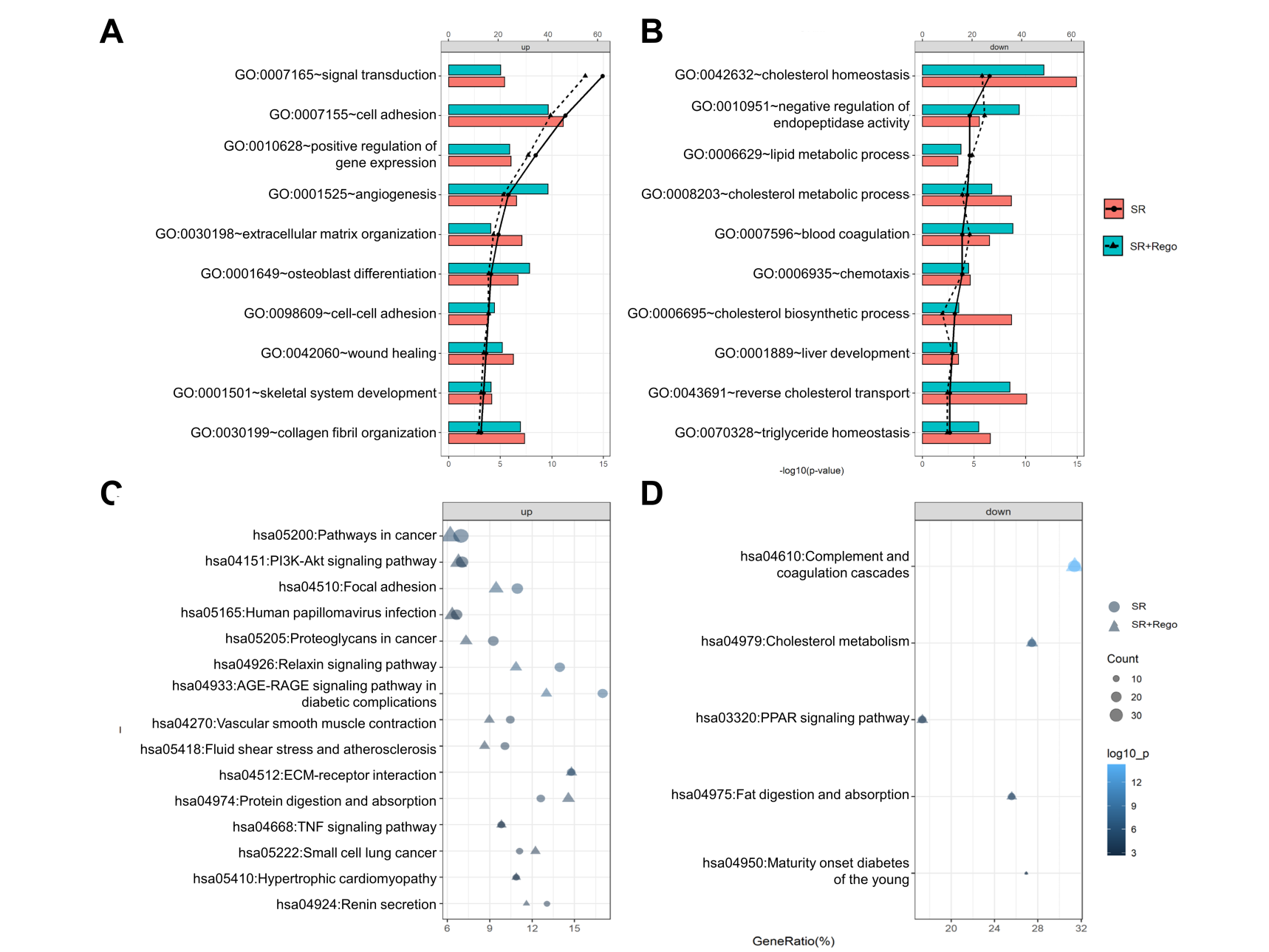

Supplement: S4 Fig — (A, B) GO term analysis of the commonly associated BPs with (A) upregulated and (B) downregulated genes. The top 10 enriched GO terms in BPs are shown, with the number of genes and p-values displayed. The column represents the count value indicating the number of genes enriched in the GO term, and the black line represents the–log10 (p-value) value. The red bar and solid black line indicate the SR cells, while the cyan bar and dotted black line indicate the SR+Rego cells. (C, D) KEGG pathway analysis of the commonly associated pathways with (C) upregulated and (D) downregulated genes. The size of the bubble represents the count value, and the color represents the p-value. Circle and triangle symbols indicate SR and SR+Rego cells, respectively. (TIFF) [file pone.0301663.s004.tiff]

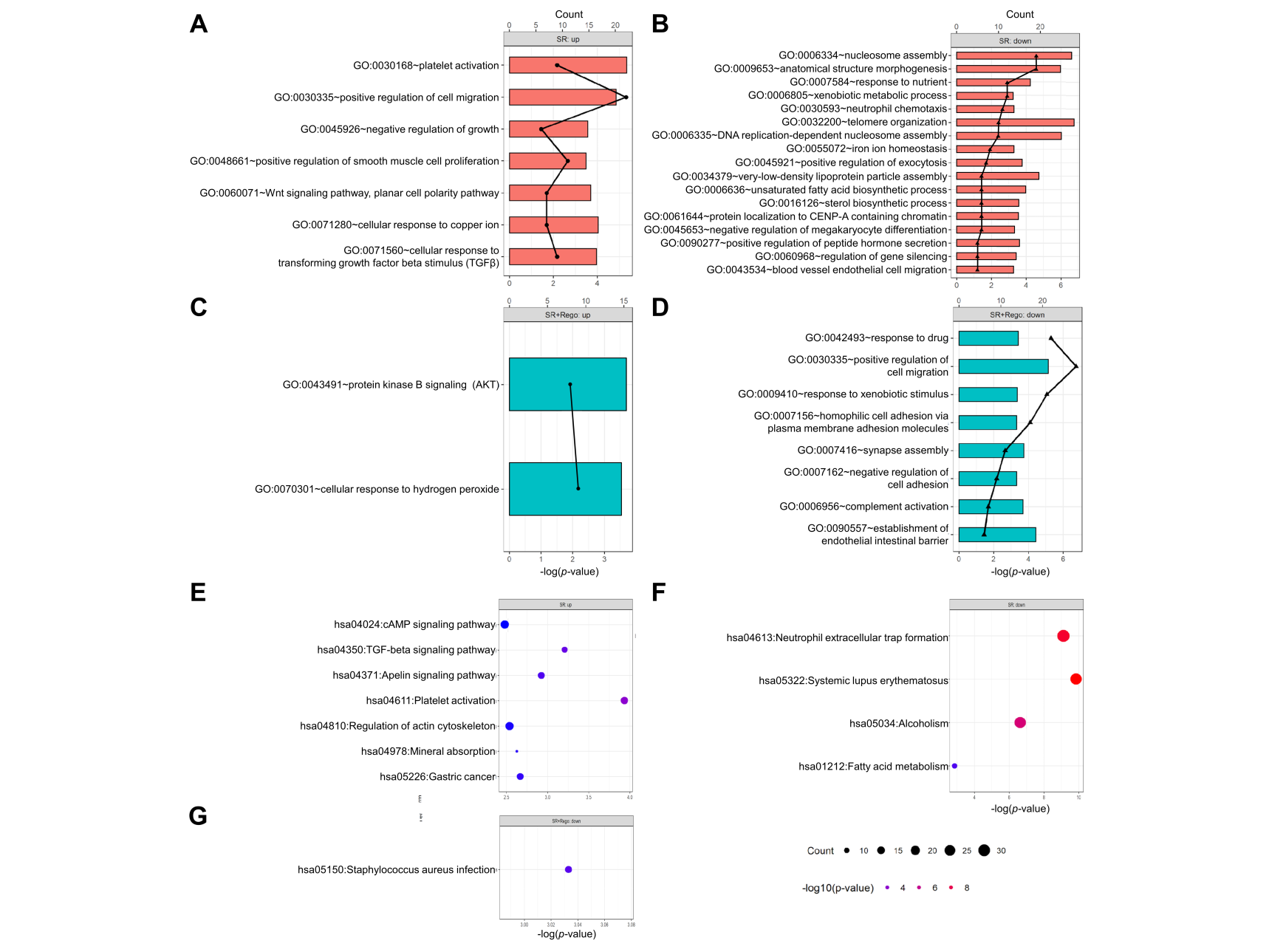

Supplement: S5 Fig — (A–D) GO term analysis of (A) upregulated genes in SR cells, (B) downregulated genes in SR cells; (C) upregulated genes in SR+Rego cells; (D) downregulated genes in SR+Rego cells. The numbers of genes and p-values are displayed for the top 10 GO terms in BP category. The column represents the count value indicating the number of genes enriched in the GO term, and the black line represents the -log10 (p-value) value. (E, F) KEGG pathway enrichment analysis of (E) upregulated and (F) downregulated genes in SR cells. (G) KEGG pathway enrichment analysis of upregulated genes in SR+Rego cells. The size of the bubble represents the count value, and the color represents the–log10 (p-value). (TIFF) [file pone.0301663.s005.tiff]

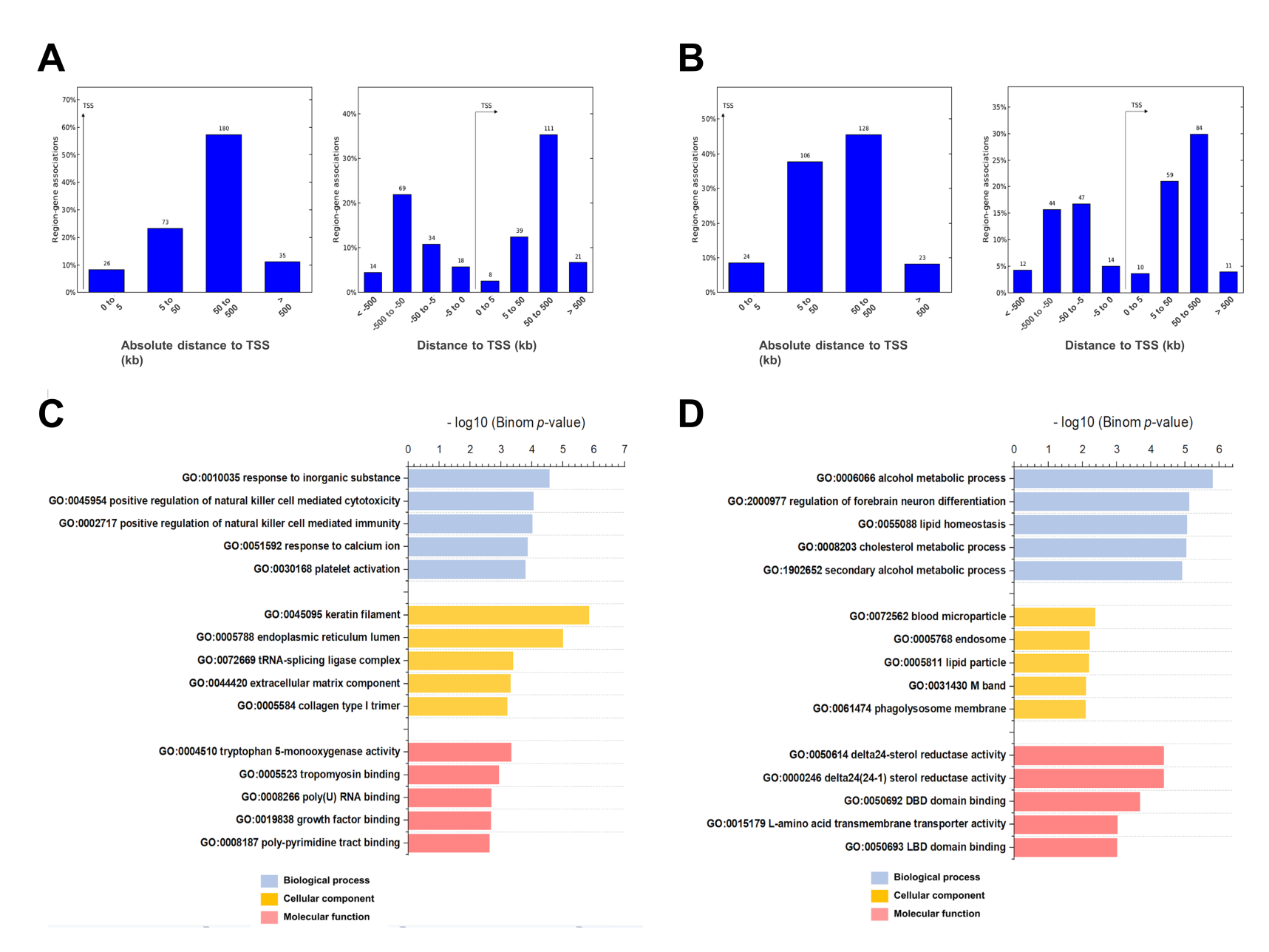

Supplement: S6 Fig — GREAT computes all GO term enrichment for genes upstream and downstream of TSS based on the genomic regions of DElncRNAs commonly expressed in SR cells. Distance (kb) to the nearest transcriptional start site (TSS) and Absolute distance to DElncRNAs and TSS sites of up- (A) and downregulated (B) lncRNAs in SR cells. Functional annotation analysis of up- (C) and downregulated (D) DElncRNAs using GREAT shows for the top 5 GO terms in BP (top panel), CC (middle panel), and MF (bottom panel). (TIFF) [file pone.0301663.s006.tiff]

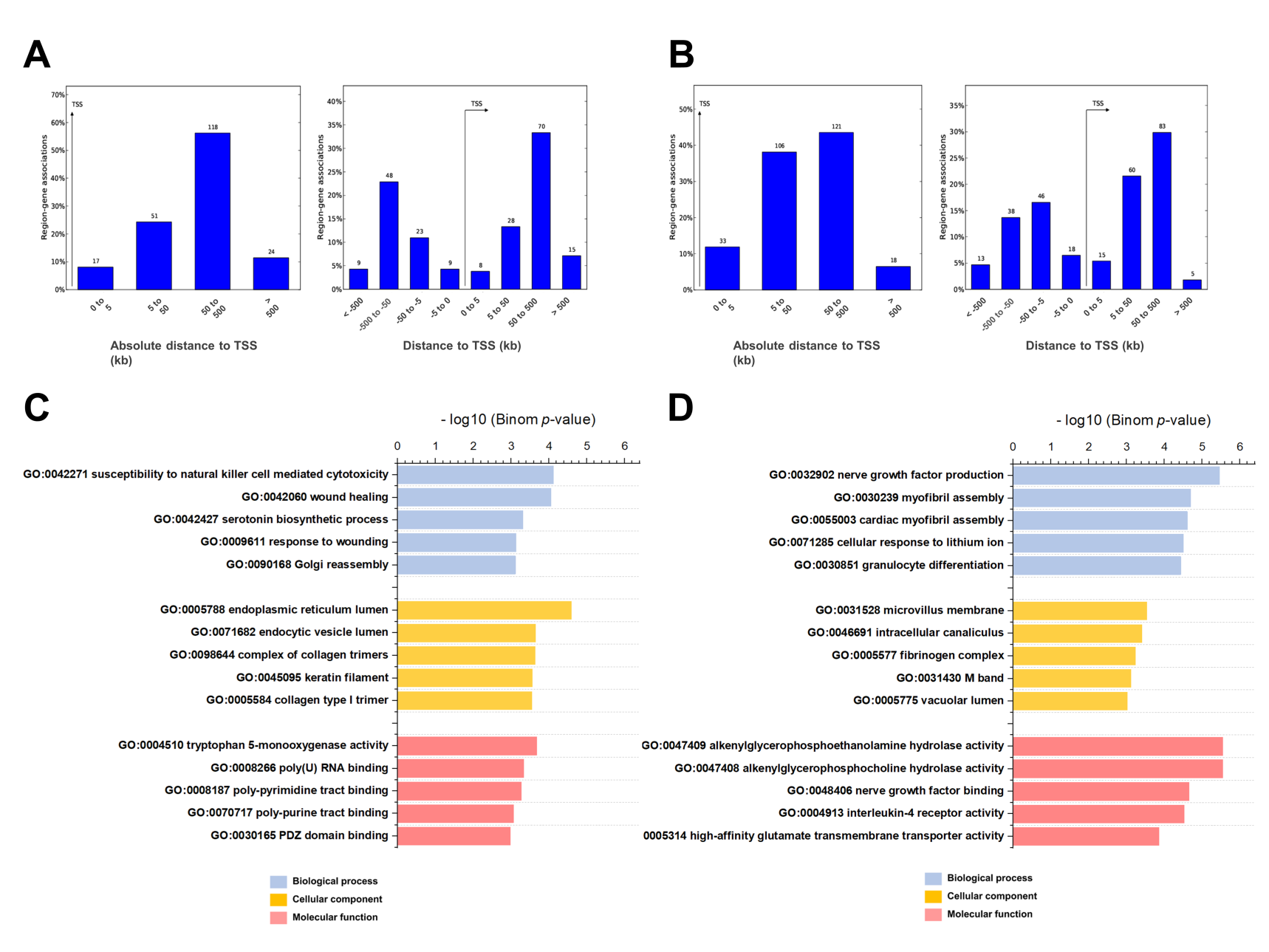

Supplement: S7 Fig — GREAT computes all GO term enrichment for genes upstream and downstream of TSS based on the genomic regions of DElncRNAs commonly expressed in SR+Rego cells. Distance (kb) to the nearest transcriptional start site (TSS) and Absolute distance to DElncRNAs and TSS sites of up- (A) and downregulated(B) lncRNAs in SR+Rego cells. Functional annotation analysis of up- (C) and downregulated (D) DElncRNAs using GREAT shows for the top 5 GO terms in BP (top panel), CC (middle panel), and MF (bottom panel). (TIFF) [file pone.0301663.s007.tiff]
